# Mapping the core senescence phenotype of primary human colon fibroblasts

**DOI:** 10.18632/aging.205577

**Published:** 2024-02-21

**Authors:** Namita Ganesh Hattangady, Kelly Carter, Brett Maroni-Rana, Ting Wang, Jessica Lee Ayers, Ming Yu, William M. Grady

**Affiliations:** 1 Translational Science and Therapeutics Division, Fred Hutchinson Cancer Center, Seattle, WA 98109, USA; 2 Public Health Sciences Division, Fred Hutchinson Cancer Center, Seattle, WA 98109, USA; 3 Division of Gastroenterology, University of Washington School of Medicine, Seattle, WA 98195, USA

**Keywords:** senescence, senescence associated secretory phenotype, SASP, colorectal cancer, cancer

## Abstract

Advanced age is the largest risk factor for many diseases and several types of cancer, including colorectal cancer (CRC). Senescent cells are known to accumulate with age in various tissues, where they can modulate the surrounding tissue microenvironment through their senescence associated secretory phenotype (SASP). Recently, we showed that there is an increased number of senescent cells in the colons of CRC patients and demonstrated that senescent fibroblasts and their SASP create microniches in the colon that are conducive to CRC onset and progression. However, the composition of the SASP is heterogenous and cell-specific, and the precise senescence profile of colon fibroblasts has not been well-defined. To generate a SASP atlas of human colon fibroblasts, we induced senescence in primary human colon fibroblasts using various *in vitro* methods and assessed the resulting transcriptome. Using RNASequencing and further validation by quantitative RT-PCR and Luminex assays, we define and validate a ‘core senescent profile’ that might play a significant role in shaping the colon microenvironment. We also performed KEGG analysis and GO analyses to identify key pathways and biological processes that are differentially regulated in colon fibroblast senescence. These studies provide insights into potential driver proteins involved in senescence-associated diseases, like CRC, which may lead to therapies to improve overall health in the elderly and to prevent CRC.

## INTRODUCTION

Advanced age is by far the highest risk factor for many diseases such as atherosclerosis, diabetes, macular degeneration, neurodegenerative diseases, and several types of cancers, including colorectal cancer (CRC) [1]. Aging and age-associated pathologies are associated with several biological and molecular alterations that affect a variety of cell processes including cellular metabolism, inflammatory responses, proteostasis, and regulation of the epigenome. One of the most common age-related phenomena seen in various tissues and across various species is the accumulation of senescent cells. Cellular senescence, as described by Hayflick, is a state of irreversible arrest of cell proliferation [2]. It is characterized by the accumulation of DNA damage, induction of p53/p21 and/or p16^INK4a^/pRB tumor suppressive pathways, elevated senescence associated Beta-galactosidase activity at pH6, senescence-associated heterochromatin foci and morphological changes such as enlargement and flattening of cells, and the increased appearance of vacuoles [3–5]. A significant feature of senescent cells, first described in 2008 by Campisi and colleagues [6], is the production of a complex senescence associated secretory phenotype (SASP) that consists of hundreds of secreted proteins including cytokines, chemokines and growth factors. As a result of the SASP, senescent cells can modulate the surrounding tissue microenvironment, promote embryonic development, remodel the extracellular matrix, enable wound healing, and activate tumor-suppressive pathways, even in young individuals [3, 7–10]. In nonpathological states, senescent cells are cleared from healthy tissues when the inciting event is resolved [3, 8–11]. However, the persistent increase in senescent cell numbers seen in tissues of the elderly appears to lead to chronic inflammation, which can induce pro-tumorigenic pathways being activated as well age-related functional decline [12–17]. *In vivo* and *in vitro* studies confirm that removal of senescent cells, and thereby reducing SASP, can prevent or slow the onset of age-related pathologies [18–20]. However, the composition of SASP is cell-specific, and heterogenous, differing based on the senescence triggering agent [21–26].

In the context of CRC, we recently showed that an increased number of senescent fibroblasts is present in the normal healthy colons of subjects with concurrent CRC [12] and hypothesized that their SASP may alter the colon tissue microenvironment to create microniches that are conducive to CRC onset and progression. Our *in vitro* studies have already demonstrated that SASP from oxidative stress-induced senescence in colon fibroblasts can activate cancer hallmark molecular pathways and behaviors such as proliferation, migration and invasion in normal epithelial cells and benign and malignant neoplastic cell lines. Besides oxidative stress, the colon tissue microenvironment is rich in several genotoxic stressors due to the presence of toxic by-products of consumed food, dysbiosis of microbiota, secreted microbial toxins, etc., which have the potential to induce a unique senescent profile and SASP’s in colon fibroblasts. The senescent signatures of various cell types and established cell lines [21, 23–28], including dermal, pulmonary, neuronal and bone marrow cells, has revealed that each cell type has a unique SASP, with some components that are common to a universal senescent cell signature and others that are unique. Notably, despite the impact of aging on colon health and colon cancer, an atlas of the SASP produced by colon fibroblasts has not been established.

To address this gap in knowledge, we utilized various relevant stressors to induce senescence in primary cultures of colon fibroblasts and perform RNA sequencing (RNASeq) to define an atlas of stressor-specific senescent profiles and a core senescent profile that is commonly regulated by all senescence inducers. We validated six of eight core senescent candidates in the transcriptome by qPCR and in the secretome by a Luminex assay. We also defined enriched pathways, biological processes and molecular functions that are characteristic of the core senescent profile in colon fibroblasts.

## MATERIALS AND METHODS

### Primary fibroblast isolation

The normal healthy rectosigmoid colon adjacent to concurrent tumor was collected during surgical resection of tumor after informed consent through the IRB approved ColoCare Study, as previously described [12]. Participants consisted of men and women aged 43 years to 71 years old (Supplementary Table 1). Each colon tissue was cut into small (1–2 mm) fragments and washed with cold DPBS three times. The tissue fragments were transferred into a 50 ml conical tube and incubated with 25 ml HBSS (without Ca^2+^ and Mg^2+^) with 5 mM EDTA at 37ºC in shaking air bath (250 rpm) for 1.5 hours to denude the fragments. After a wash in cold DPBS, the fragments were incubated with 20 ml HBSS (without Ca^2+^ and Mg^2+^) with 2000 U of collagenase D and 20 U of Dispase at 37°C in a shaking air bath (250 rpm) for 1 hour. The isolated cells were centrifuged (200 × g at 4ºC for 5 min) and resuspended with EMEM medium with 10% fetal bovine serum and 1% penicillin-streptomycin. The resuspended cells were strained through a 70 μm cell strainer and incubated in TC-treated T25 flasks at 37°C in 5% oxygen. After overnight incubation, the cells were washed gently with EMEM medium. The adherent cells include epithelial cells and fibroblasts, with only the fibroblast cells surviving the first passage. Fibroblasts were cultured for 4–6 Population Doublings (PD) prior to use for senescence induction.

### Senescence induction and detection

For senescence induction, primary human colon fibroblasts were plated on day 0 at a density of 100,000 cells/well in a 6-well plate and allowed to adhere for 24 hours. Senescence was induced in primary cultures of human colon fibroblasts using three different stressors – hydrogen peroxide, doxorubicin and bleomycin. For oxidative stress-induced senescence, cells were treated with 400 μM hydrogen peroxide (30% w/v Sigma Aldrich) for 2 hours on day 1, followed by media removal, three 30 second-washes in 1X DPBS and recovery in growth media for 2 days. This treatment was repeated on days 4 and 7. For doxorubicin induced senescence, cells were incubated with 250 mM doxorubicin for 24 hours, then media was discarded, cells were washed three times in 1X DPBS and recovered in growth media for 9 days. For bleomycin induced senescence, cells were treated with 10 ng/mL bleomycin sulphate for 3 hours. At the end of treatment, cells were washed in DPBS and recovered with media changes, as needed, until day 10. The cellular morphology was observed daily and cells were split in a 1:2 ratio cells were >90% confluent. Proliferating cells with the same number of PD’s treated with vehicle were used as control. At the end of recovery from each treatment, on day 10, cells were plated for senescence detection and harvested for RNA isolation if >80% cells were senescent. Senescence was confirmed using the commercially available senescence associated B-gal assay kit (Biovision, Milpitas, CA, USA #K320-250) as per manufacturer recommendations. Cells plated in 24-well plates were fixed and incubated overnight in X-gal solution. At the end of 16 hours of incubation, the cells were washed first with PBS, methanol at room temperature and air dried in a dark room. The number of SA-β-gal positive cells were counted using ImageJ 1.5i using the multi-point tool.

### RNA isolation and RNASeq analysis

RNA was isolated from pelleted cells using TRIzol. Briefly, cell pellets were resuspended in 2–3 mL of TRIzol and homogenized using a 3 mL syringe and a 0.8 × 25 mm gauge needle. Samples were incubated at RT for 5 mins, 0.2 mL chloroform was added to each tube and shaken vigorously. Samples were incubated at room temperature for 2–3 mins, centrifuged at 4°C for 15 mins at maximum speed. The clear aqueous layer was transferred to a clean centrifuge tube, ensuring that none of the buffy coat was not included. One microliter glycogen and 0.5 mL isopropanol were added to each tube followed by vigorous shaking. After incubation for 10 mins at 4°C, RNA was pelleted by centrifugation at maximum speed for 10 mins. The RNA pellet was washed with 75% ethanol three times. After the last wash, the pellet was dried on a heat block at 55°C for 10 mins. After cooling to room temperature, the RNA was resuspended in nuclease free water and measured using nanodrop. RNA was cleaned using sodium acetate to ensure an acceptable 260/280 ratio with 260/280 ratios more than 1.9. RNA quality was confirmed using the Agilent 4200 Tapestation. A hundred nanograms from each of the non-senescent and senescent primary colon fibroblast cells (derived from three subjects and induced into senescence using oxidative or genotoxic stress) were prepared at 10 ng/mL in nuclease free water Library preparation and RNASequencing was performed by the Genomics and Bioinformatics Core at the Fred Hutchinson Cancer Center. Libraries were prepared using TruSeq Stranded mRNA (Illumina, San Diego, CA, USA) and sequencing was performed at a depth of 30 M reads/sample with 50 paired end reads/cycle using a P2 flow cell on the NextSeq2000 Sequencing system (Illumina, San Diego, CA, USA).

### Analysis of the RNA-seq data

Quality control for raw fastq files were performed with FastQC [29] and MultiQC, the low-quality reads and 3′ adapters were trimmed with Trim Galore! [30] and Cutadapt [31]. Then the trimmed reads were aligned to reference human genome (hg38) with the RNA-seq aligner STAR [32]. Subsequently, gene expression for each sample was quantified by counting the number of read fragments that were uniquely mapped to genes using the RSEM [33]. Genes with average read count <5 were firstly filtered out. Differential expression (DE) analysis was performed using the R package DESeq2 [34], and the genes with FDR <0.01 and log_2_ (Fold Change over non-senescent cells (NS) as control cells) >1 were considered as significantly up-regulated genes.

### Pathway and gene set analyses

The enrichment of DEGs in KEGG pathways was analyzed using the R package Limma [35] and the ‘Kegga’ function while the enrichment of DEGs in GO terms was analyzed using ShinyGO v0.76. An enrichment for secreted proteins in the core senescent profile versus the total transcriptome was identified by generating the number of transcripts coding for secreted proteins in a randomly chosen set of one thousand transcripts from each of the genelists, for the total transcriptome consisting of 15569 transcripts, this was repeated a total of three times. The enrichment of secreted proteins in the core senescent profile was confirmed by Fisher’s Exact test [36].

### Data visualization

Volcano plots were visualized in R using the ‘EnhancedVolcano’ package. KEGG enrichment analysis was plotted in R using the ‘ggplot2’. The gene ratio was calculated by dividing the number of DEGs in our dataset by the total number of genes in a particular pathway. This was plotted along the x-axis with FDR adjusted *p*-value represented by color and the number of DEGs found represented by size. GO term enrichment was visualized using the ‘pheatmap’ package. Hierarchical clustering was applied to the GO term and its associated log2FC to cluster GO terms with similar log2FC. The package ‘RColorBrewer’ was used to generate color palettes.

### Synthesis of cDNA and quantitative real-time PCR analysis

RNA concentrations were measured at 260 nm using NanoDrop (Thermo Fisher Scientific, Waltham, MA, USA). The cDNA was synthesized using iScript™ cDNA Synthesis Kit (Bio-Rad, Hercules, CA, USA) according to manufacturer’s instructions. Quantitative real-time quantitative PCR was performed using predesigned primer-probe sets (Thermo Fisher Scientific) for the various transcripts, including GAPDH which was used for normalization of gene expression levels. All quantitative real-time PCR were performed via a CFX96 Touch Real-Time PCR Detection System (Bio-Rad) and results were analyzed using CFX Manager software, version 3.1 (Bio-Rad). GAPDH (pre-designed primer-probe set) was used for normalization of cDNA loading between samples and fold changes in transcript levels versus the non-senescent group were calculated by the 2^−ΔΔCt^ method.

### Luminex assay

The Luminex^®^ Multiplex Assay Customization Tool (R&D Systems, Minneapolis, MN, USA) was used to generate an assay to detect analytes GDF15, MMP3, CXCL8/IL8, CCL5/RANTES, CXCL5/ENA78, CCL20/MIP3α and CX3CL1/fractalkine, Luminex assay was performed as per manufacturer instructions. All incubations in this assay were performed at room temperature, on an orbital shaker at 800 rpm. Briefly, 50 μL of the conditioned media or provided standard was loaded in each respective well along with 50 μL of the provided microparticle cocktails. The plate was sealed and incubated, and then washed with the provided 1X wash buffer four times using a magnetic base below the plate. Subsequently, the wells were incubated with biotin and streptavidin, with washes after each step. After a final wash, the microparticles were resuspended in 100 μL of 1X wash buffer, placed on the shaker for 2 minutes, and then read on the *FLEXMAP 3D*^®^ provided by the Immune Monitoring Core of Fred Hutchinson Cancer Center Shared Resources. A 5-PL curve fit was used to plot standard graphs and analyte values were provided in pg/mL. After normalization to the corresponding cell count during media collection, fold changes in analytes compared to the NS group were calculated.

### Biostatistical analyses

GraphPad Prism 7 software (GraphPad) was used to analyze the qPCR and Luminex data to determine statistical significance. Data were expressed as the mean ± SEM. One way ANOVA was used to compare differences between groups in all experiments. *p* ≤ 0.05 was considered as significant.

## RESULTS

### Senescence induction and confirmation

**Figure 1:**
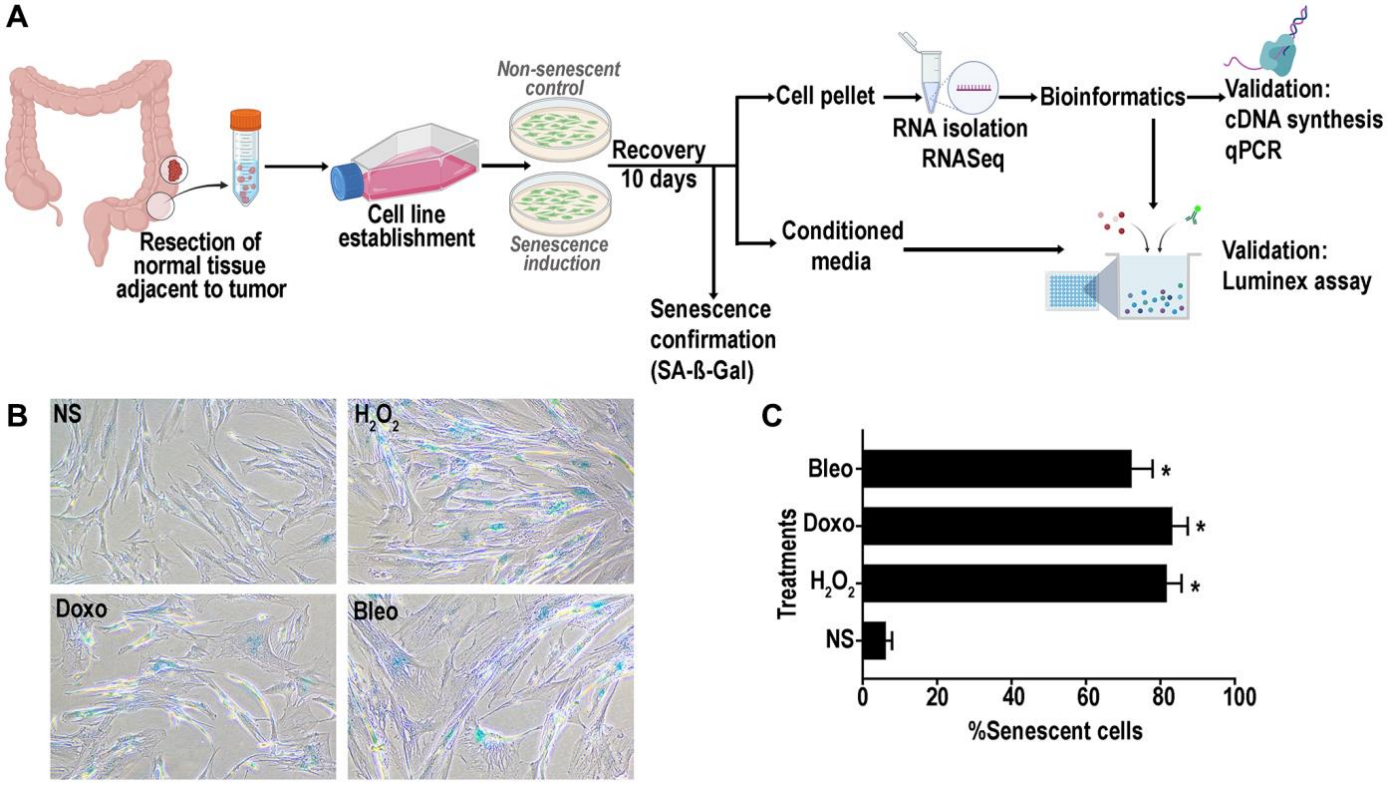
(**A**) Schematic representation of experimental design of senescence induction in the colon fibroblasts. Senescence was induced using hydrogen peroxide (H_2_O_2_, 400 μM), doxorubicin (doxo, 250 nM) or bleomycin (bleo, 10 ng/mL). Non-senescent proliferating (NS) cells were used as a control. (**B**) Representative SA- β -gal assay results at pH6 to confirm senescence induction in >70% of the cells. (**C**) Quantification of SA-β-gal positive senescent cells. (data are Mean ± SEM ^*^p < 0.01 versus NS).

We established 7 lines of primary human colon fibroblasts from resected normal healthy rectosigmoid colon from subjects with concurrent colon cancer. To induce senescence using genotoxic and oxidative stressors, we selected three different agents: doxorubicin, bleomycin and hydrogen peroxide as they induce senescence through different mechanisms: DNA alkylation and double-strand breakage; thymidine incorporation inhibition and single and double-strand DNA breakage; and oxidative stress, respectively. In addition, it is well known that the human colon mucosa is subject to exposure to alkylating and methylating agents and to oxidative stress making these agents physiologically relevant [37, 38]. A schematic representation of the overall study plan is described in Figure 1A. Following recovery for around 10 days from treatment initiation, cells displayed classic senescence features including flattened and enlarged morphology. Positive senescence associated Beta-galactosidase (SA-β-gal) activity at pH 6 (Figure 1B) was confirmed in more than 70% of the treated cells (Figure 1C) versus non-treated, proliferating control cell (hereon referred to as non-senescent (NS) controls).

### Differentially expressed senescent transcriptomes

**Figure 2:**
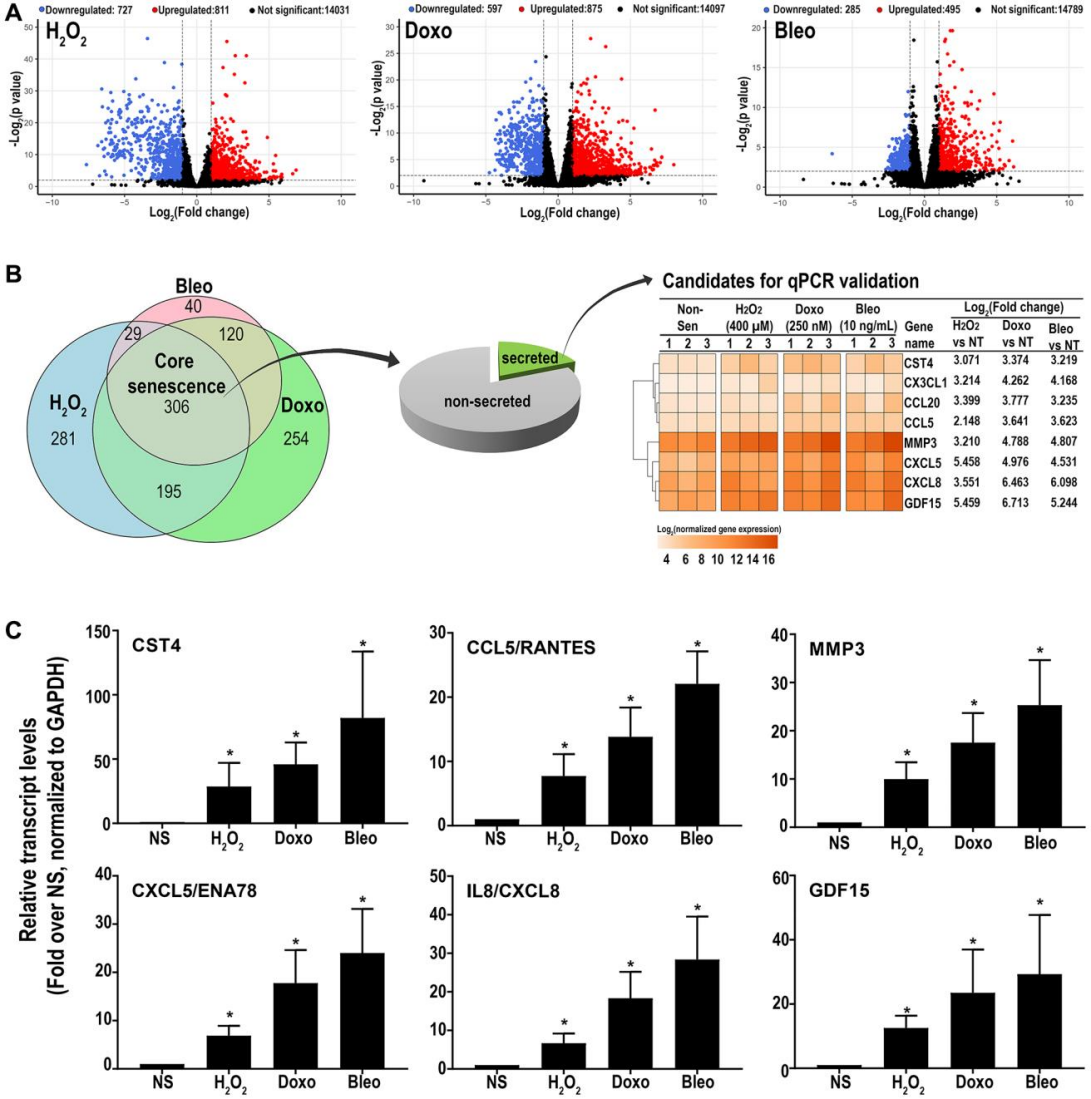
(**A**) Volcano plots of differentially expressed transcripts versus non-senescent proliferating (NS) cells (**B**) A Venn diagram showing a ‘core senescent profile’ of transcripts upregulated by all senescence inducers and heatmap of the eight candidates selected for validation at the transcriptomic level by qPCR (**C**). Data are Mean ± SEM (^*^p < 0.04 versus NS) senescent or non-senescent fibroblast lines from 7 subjects. (**C**) Quantitative RT-PCR assay results of 6 genes in the core senescence SASP demonstrates increased expression of all of the genes by any of the senescence inducing agents.

RNAseq analysis was performed on three patient-derived fibroblast lines using the HiSeq platform. Transcripts were analyzed for differential expression in senescent cells versus proliferating control cells using stringent criteria (FDR ≤0.01 and log_2_(fold change compared to NS) ≥ or ≤1). Out of 15,569 detected transcripts, several differentially regulated transcripts associated with inducer-specific senescence were found: 1538 transcripts in hydrogen peroxide-induced samples, 1492 in doxorubicin-induced samples, and 780 in bleomycin-induced samples. (Figure 2A). A Venn diagram (Figure 2B) was used to identify senescence-associated genes common to all methods of induction and is here-on referred to as the ‘core senescent profile’. The complete list of differentially expressed genes in the core senescent profile is provided in Supplementary Table 2. As a secondary confirmation of senescence, we assessed the expression of known markers of senescence and identified elevated *CDKN1A/p21^Cip1^* and *CDKN2A/p16^Ink4a^*, and decreased *LMNB1* transcripts levels compared to the NS group (Supplementary Figure 1). Since the intent of the study was to identify genes that may contribute to the colon SASP, our further analyses focused on transcripts that are up-regulated with senescence.

**Figure 3:**
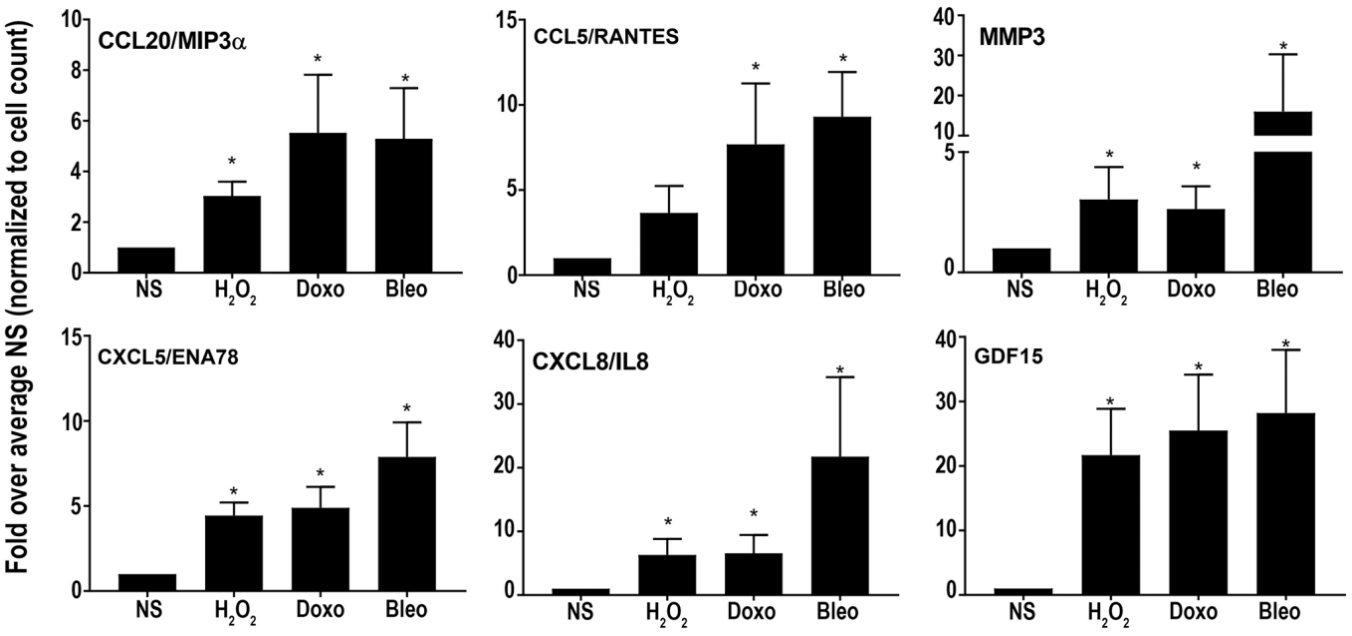
**Levels of secreted SASP candidates in the conditioned media of senescent or non-senescent fibroblast lines from at least 6 subjects.** All analytes were normalized to the cell count obtained during conditioned media collection and fold changes were calculated over respective NS group. Data are represented as Mean ± SEM (^*^p < 0.05 versus NS). NS = nonsenescent.

Overall, the core senescent profile was composed of several upregulated cytokines and chemokines (*CXCL1/GROα, CXCL2/GROβ, CXCL5/ENA78, CXCL8/IL8, CXCL14*, and *CCL2/MCP-1, CCL5/RANTES, CCL20/MIP3α*), growth factors (*GDF15, BMP2*), proteases (*MMP3, MMP12, PLAT, SERPINI1*), protease inhibitors (*CST1, CST2, CST4, C3, IGFBP2*), tumor necrosis factor receptors (*TNFSF13B, TNFRSF10C*), transmembrane proteins including transporters and channels, histones, signaling molecules (*NOTCH3, NR4A2, FOSC, RELB, STAT*), and glycoproteins such as *STC1*. We specifically assessed colon fibroblast senescence genes that could contribute to a SASP, modulate the tissue microenvironment, alter the function of the colon and potentially alter the risk for disease. We thus focused our validation studies on those transcripts that are translated to secreted proteins and focused on the core senescent profile shared by all the inducing agents, which interestingly, is enriched for secreted proteins (*p* < 0.0001). We selected eight candidates (*CST4, CCL20, CX3CL1, CXCL5, CXCL8, GDF15, CCL5* and *MMP3*) that play an important role in cancer pathogenesis for further study [3, 4, 8–12, 15, 39]. Another relevant SASP gene, *GDF15* has already been characterized for its pro-tumorigenic effects in CRC by our group [12]. We first performed RT-qPCR using transcript-specific pre-designed primer pairs to confirm the RNA-Seq results (Figure 2C). Expression levels of all transcripts were up-regulated in all senescent cells, except for *CX3CL1* and *CCL20*, which were below the detectable range in all samples. Conditioned media was then collected at the end of senescence induction to assess protein secretion (Figure 3). After normalization to the number of cells contributing to the conditioned media, we confirmed the elevated secretion of CXCL8, CCL20, CXCL5, GDF15, MMP3 and CCL5 in senescent cells, while CX3CL1 (fractalkine) was undetectable. Together, these findings confirm that elevated expression of *CXCL8, CCL20, CXCL5, GDF15, MMP3* and *CCL5* at the transcript and protein levels are part of a universal senescent colon fibroblast SASP.

### Analyses of differentially regulated pathways and gene ontologies of the colon fibroblast core senescent profile

**Figure 4:**
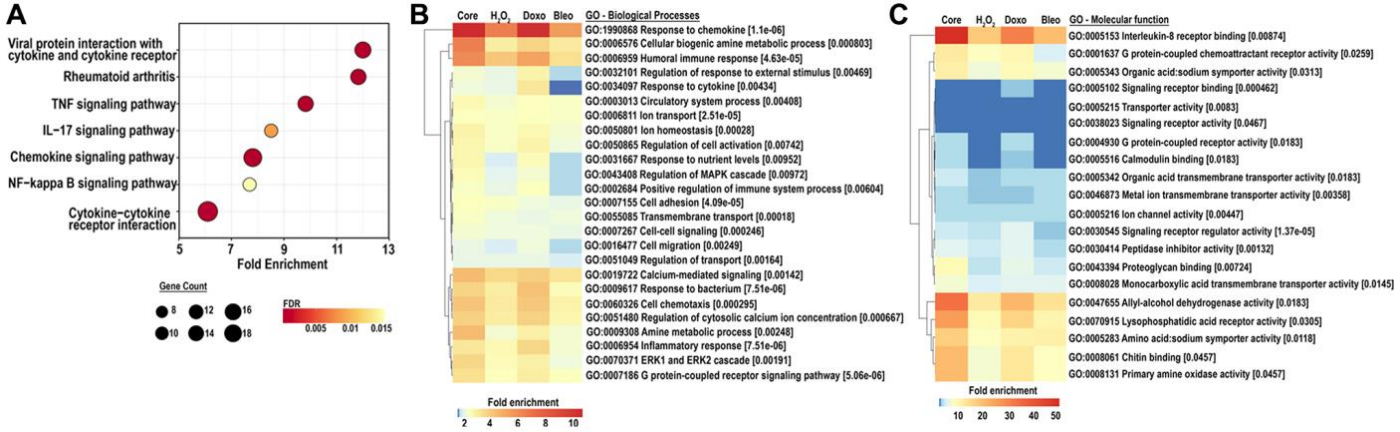
(**A**) KEGG analysis for the top 25 most enriched pathways in the core senescent profile (FDR ≤0.01). Gene counts represent the number of differentially expressed genes, color scales represent the FDR, and fold enrichment is plotted on the x-axis. (**B**, **C**) GO analyses for biological processes and molecular function enriched in the colon fibroblast core senescent profile (FDR <0.01). The top 25 entries are represented after removing redundant processes and functions.

To further investigate a shift in cellular function corresponding to the senescent associated transcriptome, we scrutinized pathways upregulated by either doxorubicin, bleomycin or H_2_O_2_-induced senescence as well as in the core senescent profile. We performed KEGG analysis for pathways enriched in stimulus-specific senescent transcriptomes as well as in the core senescent profile. The most robustly enriched pathways (Figure 4A) included the cytokine-cytokine receptor pathway and chemokine signaling pathways (list of genes in Supplementary Table 3). An enrichment of TNF pathway and NF-κB signaling pathway was found, consistent with prior studies [40]. Analyses were also performed for gene ontology based on biological processes (GOBP) and molecular functions (GOMF) of the core senescence-associated profiles, which characterized the dominant biological processes and molecular functions of cells expressing this profile. We identified 25 top pathways (Figure 4B), which were selected after accounting for redundancies by collating all relevant pathways with over 90% gene overlap (full dataset in Supplementary Table 4A, 4B). Similar to KEGG analysis, GOBP identified several cytokine-related biological processes with autocrine/paracrine significance including chemokine-mediated signaling pathway, cellular response to chemokine, and immune modulation including chemotaxis and migration of various immune cells (granulocytes, neutrophils, leukocytes and myeloid derived leukocytes). The core senescent profile also displayed the activation of several signal transduction cascades including STAT pathways (via tyrosine phosphorylation), MAPK and ERK pathways, calcium-mediated signaling pathway and G protein signaling pathways. The enrichment of transporter and channel activity, and calcium-ion regulatory mechanisms was also observed, indicating ionic flux in senescent cells. Analyses of molecular function (Figure 4C, Supplementary Table 5) similarly indicated the enrichment of chemokine-related activity, particularly that of CXCL8/IL8, inflammatory mechanisms, elevated activity of various transmembrane channels, activation of receptors to chemokines/cytokines and enzyme lytic activity, indicating an ability to modulate the surrounding milieu. Thus, KEGG, GOBP and GOMF together confirm a persistent elevation in cytokines and chemokines that may have paracrine and systemic effects on the tissue microenvironment. Interestingly, our analyses did not identify any notable differences in pathways or GO’s related to cancer or aging between the various treatments for senescence induction.

## DISCUSSION

The role of senescence in pathophysiology has been well documented, as has the vast heterogeneity in the senescent profile across cell types [21–26]. Several senescence databases have been created including Cellular Senescence Network (SenNet), SeneQuest by the International Cell Senescence Association, CellAge [41] at the Human Ageing Genomic Resources, and the SASPAtlas [21]. While these databases serve as excellent resources to explore senescent phenotypes across multiple cell types, human colon fibroblasts have not been described in any of them. Our study fills in this knowledge gap by describing the senescent phenotype of primary human colon fibroblasts. Our study is of particular value because it informs our understanding of senescence in colon health and disease and the impact of secreted senescence products on shaping the tissue microenvironment in the colon. Using RNASeq, we generated an atlas of the senescent associated secretory phenotype of human colon fibroblasts after treatment with different classes of physiologically relevant senescence inducers and identified a core SASP profile that is shared by all the inducers. We used primary adult colon fibroblast lines to generate senescence profiles that are most likely to reflect the SASP of human *in vivo* fibroblasts. For a subset of the SASP genes, we confirmed that the senescence-associated transcripts do ultimately result in secreted proteins. We also identified key pathways and biological processes that are differentially regulated in colon fibroblast senescence through KEGG and GOBP/GOMF analysis.

Of interest, we identified a set of SASP proteins secreted by colon fibroblasts that are biologically plausible factors for affecting the health and disease of the colon in people. Notably, these SASP proteins include GDF15, CXCL5, CXCL8/IL8, CCL5/RANTES, and MMP3. Their impact in causing aging-associated changes such as inflammation, aging-related pathologies and in cancer mechanisms have also been previously demonstrated in mouse models and cell line systems. GDF15, a distant member of the transforming growth factor (TGF)-β superfamily, was initially recognized as macrophage inhibitory cytokine-1 (MIC-1) and noted for its ability to suppress various immune cells including other macrophages, dendritic cells, neutrophils, T cells and natural killer cells [42]. More recently, the plasma GDF15 level has been associated with age and is predictive of mortality and multimorbidity [43]. It signals via the GFRAL receptor [44] and has been shown to promote cancer hallmark behaviors in colon adenocarcinoma cells [12]. GDF15 is also a highly expressed SASP component in senescent dermal and pulmonary fibroblast and some epithelial lines that have been induced into senescence by a variety of methods [21–23].

It is notable that our core senescent profile includes chemokines of the CXCL- and CCL- families, including CXCL1, CXCL2, CXCL5, CXCL8, CXCL14, CCL2, and CCL20, have similarly been identified as SASP components in our studies and in previously published studies [15, 26]. CXCL8/IL8 is a well-studied chemokine in multiple cancer types, including colorectal, esophageal, bladder, gastric, breast, and others [45]. CXCL8 acts as a strong chemoattractant, potently increasing tumor-promoting behaviors including tumor proliferation, migration, invasion, anoikis suppression, an epithelial to mesenchymal transition (EMT) [39, 46–49]. CXCL8 can also suppress immune responses in the surrounding microenvironment [45, 50, 51]. Recent evidence suggests that increasing serum CXCL8 levels can predict resistance to anti-PD-1 immunotherapy in non-small-cell lung cancer patients, which has led to the development of targeted therapies directed at CXCL8 (e.g., HuMax-IL8) for the treatment of some types of cancers [52, 53]. Besides its role in cancer, CXCL8, along with CXCL1, 2, and 5 are noted for their autocrine and/or paracrine effects in reinforcing senescence. This occurs when these chemokines bind the CXCR2 receptor which leads to the downstream activations of the NF-kB signaling pathway [54]. CCL20 has similarly also been noted for reinforcing a senescence phenotype [4, 55]. Of note, these SASP cytokines can further propagate senescence in neighboring cells, thus shaping a broader senescent tissue microenvironment [4, 55, 56].

In addition to GDF15 and the chemokines and cytokines we observed in the colon fibroblast SASP, we also identified several other SASP proteins that may mediate health and disease in the colon *in vivo*. Matrix metalloproteinases 3 and 12 (MMP3 and 12), which are elevated in the colon core senescent phenotype, have also been also reported in previously described senescent cell line studies and are robustly elevated in senescent murine tissue [57, 58]. Interestingly, the elevation of several well-reported [15] senescence-induced targets including *IL1A, IL1B, IL33*, and *IL6* was observed to be senescence inducer specific in colon cells and thus were excluded from the core senescent profile, reflecting the heterogeneity in senescence that results from different etiologies.

Our KEGG and GO analyses highlighted the cytokine and chemokine mediated events, including the TNF pathway and NF-κB signaling pathway. Besides being amplified by autocrine effects of cytokines, the TNF and NF-κB pathways have also been frequently associated with senescence and aging [40]. In the colon fibroblasts, the senescence associated NF-κB pathway appeared to be driven through the canonical mechanism (involving *cIAP1/2* or *BIRC2, CXCL8* and *VCAM1*) as well as non-canonical mechanisms (involving *BAFF, CD40*, and *RELB*) (Supplemental Table 3). Germane to our findings, senomorphic therapies that prevent the production of the SASP, may do so by inhibiting the NF-kB pathway directly (e.g., resveratrol, metformin, etc.,) or indirectly through mTOR inhibition (e.g., rapamycin). These agents are under investigation for their ability to minimize or prevent age-related and chronic pathologies [59]. Interestingly we also observed an enrichment of pathways associated with rheumatoid arthritis and atherosclerosis, both of which are age-associated and inflammatory diseases. GOBP and GOMF analyses of the SASP subset of the core senescent profile are suggestive of paracrine processes include extracellular matrix disassembly, organization, extracellular structure organization and collagen catabolic process (Supplementary Table 6A, 6B). Indeed, these observations support the hypothesis that senescent cell accumulation secondary to increased oxidative stress, exposure to DNA alkylating agents, or age can create an environment with persistent elevation in pro-inflammatory cytokines and chemokines, that can eventually lead to modulation of the surrounding matrix and chronic activation of inflammatory processes [3, 4, 8, 10, 11, 15, 60].

It is notable that our results have similarities to a variety of other senescent associated datasets. Firstly, we compared colon fibroblast senescence transcriptome to a human aging secretome [61]. We found that *GDF15, CXCL1, CXCL8, CXCL14, CCL5*, and several other core senescence candidates (Supplementary Table 7), overlap with aging-related secretome, as previously described. We next assessed commonalities of the colon fibroblast senescent phenotype with previously described phenotypes of senescent fibroblasts in tissues other than the colon. We focused on a large proteomic study and meta-analysis on the SASP of pulmonary fibroblasts induced into senescence by irradiation, oncogene activation and atazanavir treatment [21]. The genes upregulated in all three conditions that overlapped the colon core senescence included *AKR1B1, IGFBP2, C3, CXCL1, STC1* and *GDF15* (FDR <0.01). Of these, *GDF15* was consistently upregulated in a ‘universal senescent profile’ of various epithelial and fibroblast cell lines. We also found that doxorubicin-induced-senescence of pulmonary fibroblasts [22] shared 61% of the colon doxorubicin-induced transcripts, including the validated core colon senescence candidates *CCL20, CXCL5, CST4, GDF15, MMP3*, as well as other targets including *AKR1B1, IGFBP2, C3, CXCL1*, and *STC1*. Lastly, we compared our results with those in the SenMayo gene set, a physiologically relevant senescence gene set [26], which is curated to accurately identify senescent cells with aging across tissues and species. We found that that nearly a quarter of the SenMayo list overlaps with the core senescent colon fibroblast profile, and of these, four genes (*GDF15, CXCL1, C3* and *IGFBP2)* are also common to the Basisty universal fibroblast senescent signature [21]. In aggregate, our observations support the concept that senescence-associated profiles have several commonalities among cell types but are also very heterogenous and specific to cell type and method of induction.

We similarly compared the phenotype of senescent human colon fibroblasts to that of cancer-associated fibroblasts (CAFs), which occur in the immediate tumor microenvironment. Similar to senescent fibroblasts, CAFs are large in size and have indented nuclei. They secrete a variety of growth factors, chemokines, extracellular matrix (ECM) proteins and well as ECM-degrading metalloproteases, which have been implicated in promoting oncogenic behaviors of cancer and in resistance to anti-cancer therapies [62–69]. Evidence also supports a role for senescent CAFs in tumor progression [70–72]. Overall, a large similarity has been observed in oncogenic secretions from CAFs and senescent non-cancer cells. Single cell sequencing has shown that CAFs are heterogenous in origin and can be functionally categorized as inflammatory, complement presenting, myofibroblastic and antigen presenting [63, 73–77]. Specifically relevant to the CRC microenvironment and senescence are the subsets of CAF subsets described by Pelka et al. (2021) that exhibited elevated levels of an inflammatory program, including genes *MMP1, MMP3, CXCL8* and *CXCL1*, which are colon fibroblast SASP components observed in our study. Additional SASP components observed in our studies (*CXCL14*, *BMP4*, *HSD17B2*, *CCL13*, *TCF21*, *SOD2*, and *F3*) formed the characterizing features of various other CAF subsets in this same study [74]. Thus, similar to CAFs, human colon fibroblast SASP can be speculated to have a variety of roles in modulating the surrounding niche in colon pathophysiology and perhaps inducing oncogenic effects in the colon.

Finally, we wish to note that although our studies have many strengths, they also have limitations. These limitations include a significant degree of subject-based heterogeneity in senescence within the same cell type that is independent of the senescence-induction regimen used, assessment of the SASP at only one time point after induction by the senescence inducing agent, and no assessment of single cell responses to the SASP inducing agent.

In conclusion, we have defined a core senescent transcriptome and SASP for primary human colon fibroblasts, validated a subset of important SASP associated secreted proteins, and described noteworthy SASP mediated changes in pathways, biological processes, and molecular functions. Based on our analyses, we propose that elevated expression *GDF15, MMP3, CXCL8, CXCL1, CXCL5, STC1* and *CCL5* are a core set of SASP genes of senescent colon and non-colon fibroblasts, which may be of use for identifying senescent cells in tissues. Our studies have also helped inform the candidate driver proteins involved in senescence-associated diseases like CRC These candidates might also prove worthy targets for anti-senescence therapies, which is a class of therapy currently under investigation for the prevention of age-related disease in people. Further studies will be needed to address the limitations of our study and to translate our understanding of the SASP and disease into clinical care.

## Supplementary Materials

Supplementary Figure 1

Supplementary Tables 1, 3, 5 and 7

Supplementary Table 2

Supplementary Table 4A

Supplementary Table 4B

Supplementary Tables 6A and 6B
