# Supplementary material for: Mapping the core senescence phenotype of primary human colon fibroblasts
Source: Aging (Albany NY). 2024 Feb 21;16(4):3068–87. doi: 10.18632/aging.205577 (PMC10929841; doi:10.18632/aging.205577)
Supplement: Supplementary Figure 1 [file aging-16-205577-s001.pdf]

SUPPLEMENTARY FIGURE

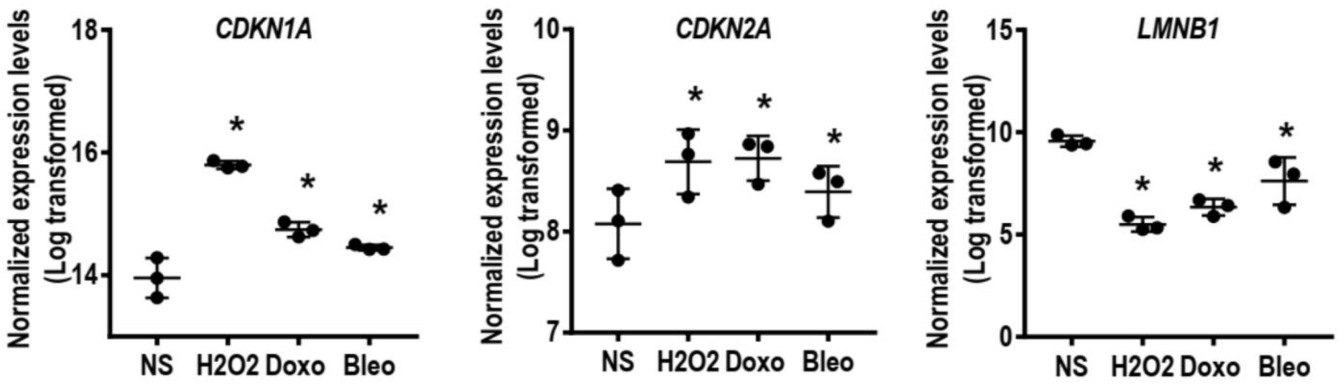

Supplementary Figure 1. Changes in expression of *CDKN1A, CDKN2A* and *LMNB1,* which are markers of senescence. Data represented are normalized gene expression values obtained from RNA sequencing data of non-senescent and senescent fibroblast lines from three subjects. *FDR≤0.001 versus NS using One-way ANOVA with Benjamini and Hochberg correction for false discovery rate.
