# Supplementary material for: Mapping the core senescence phenotype of primary human colon fibroblasts
Source: Aging (Albany NY). 2024 Feb 21;16(4):3068–87. doi: 10.18632/aging.205577 (PMC10929841; doi:10.18632/aging.205577)
Supplement: Supplementary Tables 1, 3, 5 and 7 [file aging-16-205577-s002.pdf]

SUPPLEMENTARY TABLES

Supplementary Table 1. Patient demographics for subject-derived fibroblast cell lines.

Cell Line#	Colon Location	Subject Age at time of tissue collection	Gender	Race	BMI	Smoking	NSAID	Diabetes	Use in study
NWBT3	Colon-Transverse	57	Male	White	33.67	Never	No	No	Validation by qPCR and Luminex
NWBT4	Sigmoid Colon	71	Male	White	35.61	Former	Yes	No	Validation by qPCR and Luminex
NWBT6	Sigmoid Colon	43	Male	Native Hawaiian/ Pacific Islander	32.74	Never	No	Type II	RNA Sequencing and validation by qPCR and Luminex
NWBT8	Sigmoid Colon	43	Female	American Indian/Alaskan Native	47.72	Never	No	No	RNA Sequencing and validation by qPCR and Luminex
NWBT9	Rectosigmoid colon	55	Female	White	22.37	Former	NA	pre-diabetic	Validation by qPCR and Luminex
NWBT10	Sigmoid Colon	37	Male	White	25.9	Never	N/A	No	RNA Sequencing and validation by qPCR and Luminex
NWBT14	Colon	44	Female	White	19.8	Former	Yes	No	Validation by qPCR and Luminex

Supplementary Table 3. KEGG analysis of the core senescence profile (FDR≤0.05).

Pathway	*n*	count	*p*-value	fdr	gene_ratio	pathway_id	genes
Lipid and atherosclerosis	215	11	8.17E-04	0.035859	5.116279	path:hsa05417	CYP1A1, CXCL1, CXCL2, CXCL8, MMP3, CCL2, CCL5, SOD2, VCAM1, CD36, CD40
Cytokine-cytokine receptor interaction	295	18	1.45E-06	1.27E-04	6.101695	path:hsa04060	TNFSF13B, CCR7, CXCL1, CXCL2, CXCL8, LIF, ACKR3, CCL2, CCL5, CCL20, CXCL5, CX3CL1, BMP2, TSLP, TNFRSF10C, GDF15, CXCL14, CD40
NF-kappa B signaling pathway	104	8	3.02E-04	0.015155	7.692308	path:hsa04064	TNFSF13B, CXCL1, CXCL2, BIRC3, CXCL8, RELB, VCAM1, CD40
Chemokine signaling pathway	192	15	5.38E-07	6.29E-05	7.8125	path:hsa04062	ADCY8, CCR7, SHC2, GRK4, CXCL1, CXCL2, CXCL8, SHC4, CCL2, CCL5, CCL20, CXCL5, CX3CL1, STAT1, CXCL14
IL-17 signaling pathway	94	8	1.50E-04	0.00877	8.510638	path:hsa04657	FOSB, CXCL1, CXCL2, CXCL8, MMP3, CCL2, CCL20, CXCL5
Epithelial cell signaling in Helicobacter pylori infection	70	6	9.92E-04	0.038676	8.571429	path:hsa05120	ATP6V0D2, CXCL1, CXCL2, CXCL8, F11R, CCL5
TNF signaling pathway	112	11	2.07E-06	1.45E-04	9.821429	path:hsa04668	CXCL1, CXCL2, BIRC3, LIF, MMP3, CCL2, CCL5, CCL20, CXCL5, CX3CL1, VCAM1
Malaria	50	5	0.001321	0.046365	10	path:hsa05144	CXCL8, CCL2, VCAM1, CD36, CD40
Rheumatoid arthritis	93	11	3.13E-07	5.50E-05	11.82796	path:hsa05323	TNFSF13B, CTSK, ATP6V0D2, CXCL1, CXCL2, CXCL8, MMP3, CCL2, CCL5, CCL20, CXCL5
Viral protein interaction with cytokine and cytokine receptor	100	12	7.53E-08	2.64E-05	12	path:hsa04061	CCR7, CXCL1, CXCL2, CXCL8, ACKR3, CCL2, CCL5, CCL20, CXCL5, CX3CL1, TNFRSF10C, CXCL14

Supplementary Table 5. GO analysis for molecular function on the core senescence profile (FDR ≤ 0.01).

GO ID	Pathway	Fold enrichment	Enrichment FDR	N genes	Pathway genes	Gene ratio	Genes
GO:0030545	Signaling receptor regulator activity	3.99	0.0000137	21	629	3.339	CX3CL1 TG CXCL2 TNFSF13B CCL2 CCL20 BMP2 LIF FGF13 GDF15 GPNMB TSLP CXCL14 STC1 CXCL5 CXCL1 CXCL8 LYNX1 CDNF CCK CCL5
GO:0005102	Cytokine/chemokine receptor binding	2.11	0.0004620	40	1722	2.323	CX3CL1 TG CXCL2 TNFSF13B PLAT CCL2 CPE CCL20 ITGB6 STAT1 IGFBP2 C3 BMP2 FGL2 LIF FGF13 SHC2 GDF15 CD36 GPNMB GABARAPL1 TSLP CXCL14 F11R STC1 SQSTM1 SHANK1 VCAM1 CXCL5 CXCL1 TAC3 BDKRB2 NSG1 CXCL8 LYNX1 CDNF PRKN SHC4 CCK CCL5
GO:0030414	Peptidase inhibitor activity	5.12	0.0013185	10	204	4.902	BIRC3 CST4 RENBP C3 SNCA SERPINI1 SPINT1 CST2 CST1 PROS1
GO:0046873	Metal ion transmembrane transporter activity	3.03	0.0035773	16	454	3.524	SLC4A7 SLC6A15 TMEM38A SLC1A2 SLC6A12 KCNJ2 KCNC3 KCNK1 KCNJ6 KCNH6 SCN4B SCN5A KCNK12 TPCN1 RYR2
GO:0005216	Ion channel activity	3.05	0.0044680	15	459	3.268	TMEM38A P2RX6 KCNJ2 KCNC3 KCNK1 CLCA2 KCNJ6 KCNH6 SCN4B SCN5A KCNK12 TPCN1 RYR2 TMEM150C
GO:0043394	Proteoglycan binding	8.65	0.0072366	5	38	13.158	CFH GPNMB CTSK CTSS HPSE2
GO:0005215	Transporter activity	1.92	0.0082971	31	1364	2.273	SLC4A7 SLC6A15 TMEM38A SLC7A8 P2RX6 ATP8B4 SLC16A6 SLC1A2 SLC6A12 CNNM1 KCNJ2 ABCC4 KCNC3 CD36 KCNK1 SLCO2B1 CLCA2 SLC44A3 SLC2A12 ATP6V0D2 ABCA9 KCNJ6 KCNH6 SCN4B SCN5A KCNK12 TPCN1 APOD RYR2 TMEM150C
GO:0005153	Interleukin-8 receptor binding	50.18	0.0087442	2	3	66.667	CX3CL1 CXCL8
GO:0005283	Amino acid:sodium symporter activity	16.73	0.0117724	3	14	21.429	SLC6A15 SLC1A2 SLC6A12
GO:0008028	Monocarboxylic acid transmembrane transporter activity	6.78	0.0144993	5	59	8.475	SLC16A6 SLC6A12 ABCC4 CD36 SLCO2B1
GO:0005416	Amino acid:cation symporter activity	15.05	0.0144993	3	18	16.667	SLC6A15 SLC1A2 SLC6A12
GO:0004930	G protein-coupled receptor activity	3.02	0.0182516	11	1026	1.072	CELSR3 LPAR2 ADGRB2 CCR7 ACKR3 BDKRB2 GPRC5C LPAR3 CMKLR1 ADGRB1 ADGRL1
GO:0005516	Calmodulin binding	3.20	0.0182516	10	208	4.808	DAPK2 MAP2 WFS1 IQGAP2 OBSCN ADCY8 RRAD CAMK1D SCN5A RYR2

GO:0047655	Allyl-alcohol dehydrogenase activity	33.45	0.0182516	2	4	50.000	AKR1B1 AKR1B10
GO:0005342	Organic acid transmembrane transporter activity	3.79	0.0182791	8	161	4.969	SLC6A15 SLC7A8 SLC16A6 SLC1A2 SLC6A12 ABCC4 CD36 SLCO2B1
GO:0001637	G protein-coupled chemoattractant receptor activity	11.58	0.0259279	3	26	11.538	CCR7 ACKR3 CMKLR1
GO:0070915	Lysophosphatidic acid receptor activity	25.09	0.0304585	2	5	40.000	LPAR2 LPAR3
GO:0005343	Organic acid:sodium symporter activity	10.75	0.0312890	3	28	10.714	SLC6A15 SLC1A2 SLC6A12
GO:0008061	Chitin binding	20.07	0.0457024	2	8	25.000	OVGP1 CHI3L1
GO:0008131	Primary amine oxidase activity	20.07	0.0457024	2	6	33.333	AOC2 VCAM1
GO:0038023	Signaling receptor activity	1.82	0.0467353	23	1940	1.186	CELSR3 LPAR2 NOTCH3 GPC4 P2RX6 CD40 ITGB6 NR4A3 ADGRB2 CCR7 CD36 LEF1 ACKR3 NR4A2 BDKRB2 GPRC5C LPAR3 TNFRSF10C CMKLR1 ADGRB1 ROBO2 L1CAM ADGRL1

Supplementary Table 7. Comparison of the core colon fibroblast senescence profile to an aging associated secretome described by Lehallier et al. (2019).

Gene symbol	ENSEMBL ID	H2O2 Induced Senescence vs. NS		Doxo - Induced Senescence vs. NS		Bleo - Induced Senescence vs. NS		Data from Lehallier et al. 2019		
		Average Log2FC	Adjusted p	Average Log2FC	Adjusted p	Average Log2FC	Adjusted p	p.Age	q.Age	Coefficient. Age
ADSSL1	ENSG00000185100	2.159	8.659E-16	1.591	1.714E-08	1.663	1.187E-08	7.998E-14	7.670E-13	0.0019
C1QTNF1	ENSG00000173918	1.286	1.551E-09	1.569	2.063E-13	1.746	5.610E-16	6.924E-13	6.175E-12	0.0006
CCL5	ENSG00000271503	2.148	4.320E-03	3.641	1.862E-07	3.623	6.261E-07	6.808E-09	4.425E-08	0.0017
CD36	ENSG00000135218	2.155	3.529E-08	2.520	1.151E-10	1.762	3.537E-05	2.628E-32	6.570E-31	0.0018
CDNF	ENSG00000185267	2.475	2.186E-06	2.149	7.549E-05	1.874	1.368E-03	1.264E-11	1.022E-10	0.0008
CHI3L1	ENSG00000133048	2.245	2.173E-07	1.949	1.280E-05	2.310	4.067E-07	1.695E-35	4.677E-34	0.0051
CXCL1	ENSG00000163739	1.606	6.344E-04	2.436	8.507E-08	3.009	6.272E-11	6.191E-06	2.790E-05	0.0009
CXCL14	ENSG00000145824	2.218	8.181E-06	2.738	2.307E-08	2.194	3.307E-05	1.321E-02	3.029E-02	0.0004
CXCL8	ENSG00000169429	3.214	1.242E-03	4.262	1.194E-05	4.168	4.430E-05	6.932E-08	3.937E-07	0.0008
DAPK2	ENSG00000035664	1.852	4.034E-05	1.916	2.512E-05	1.568	1.517E-03	1.376E-03	4.004E-03	0.0009
GABARAPL1	ENSG00000139112	1.057	4.308E-04	1.056	5.056E-04	1.008	1.624E-03	2.010E-10	1.484E-09	0.0016
GDF15	ENSG00000130513	3.399	1.335E-04	3.777	2.136E-05	3.235	6.352E-04	2.333E-252	1.706E-249	0.0053
GPNMB	ENSG00000136235	1.818	4.733E-38	1.034	2.892E-12	1.114	1.843E-13	1.486E-05	6.335E-05	0.0007
HK2	ENSG00000159399	1.202	2.245E-03	1.201	2.501E-03	1.249	2.593E-03	3.369E-03	9.058E-03	0.0013
L1CAM	ENSG00000198910	2.268	1.437E-07	1.812	5.562E-05	1.343	6.625E-03	3.875E-05	1.524E-04	0.0005
