# Supplementary material for: Mapping the core senescence phenotype of primary human colon fibroblasts
Source: Aging (Albany NY). 2024 Feb 21;16(4):3068–87. doi: 10.18632/aging.205577 (PMC10929841; doi:10.18632/aging.205577)
Supplement: Supplementary Table 2 [file aging-16-205577-s003.docx]

**Supplementary Table 2. Core senescence profile in senescent primary human colon fibroblasts (Log2FoldChange≥1, FDR ≤ 0.01) after different oxidative and genotoxic stressors (versus non-senescent fibroblasts). Log2FoldChange values represent the average of primary lines derived from three different subjects.**

| **Gene Symbol** | **Ensembl gene ID** | **H_2_O_2_ induced senescence** | | **Doxo-induced senescence** | | **Bleo-induced senescence** | |
| --- | --- | --- | --- | --- | --- | --- | --- |
|  |  | **Log_2_FoldChange** | **Adj p value** | **Log_2_FoldChange** | **Adj p value** | **Log_2_FoldChange** | **Adj p value** |
| ABCA9 | ENSG00000154258 | 2.5650 | 2.8395E-03 | 3.4275 | 3.9166E-05 | 3.0551 | 5.6612E-04 |
| ABCC4 | ENSG00000125257 | 1.0226 | 6.4525E-06 | 1.0338 | 6.4031E-06 | 1.0513 | 1.0678E-05 |
| ACER2 | ENSG00000177076 | 2.1121 | 1.0202E-13 | 1.5679 | 1.9439E-07 | 1.2361 | 1.7477E-04 |
| ACKR3 | ENSG00000144476 | 2.0771 | 2.2551E-08 | 1.1347 | 5.0038E-03 | 1.6065 | 7.2814E-05 |
| ADAM28 | ENSG00000042980 | 2.8826 | 2.6379E-06 | 3.0903 | 5.1754E-07 | 2.7421 | 3.1120E-05 |
| ADCY8 | ENSG00000155897 | 5.8392 | 3.0853E-03 | 6.5945 | 7.2090E-04 | 6.1717 | 2.8801E-03 |
| ADGRB1 | ENSG00000181790 | 2.1765 | 8.3067E-03 | 2.3450 | 4.3212E-03 | 2.4135 | 5.1286E-03 |
| ADGRB2 | ENSG00000121753 | 1.3481 | 3.4490E-04 | 1.2575 | 1.0712E-03 | 1.3250 | 9.2576E-04 |
| ADGRL1 | ENSG00000072071 | 1.1276 | 1.3433E-05 | 1.4665 | 8.8867E-09 | 1.2267 | 6.2465E-06 |
| ADSSL1 | ENSG00000185100 | 2.1586 | 8.6588E-16 | 1.5909 | 1.7142E-08 | 1.6629 | 1.1870E-08 |
| AKAP6 | ENSG00000151320 | 1.2166 | 1.9716E-04 | 1.8807 | 1.4866E-09 | 1.5205 | 5.7230E-06 |
| AKR1B1 | ENSG00000085662 | 1.2020 | 4.6387E-04 | 1.1156 | 1.4415E-03 | 1.3956 | 9.1689E-05 |
| AKR1B10 | ENSG00000198074 | 2.5039 | 8.5470E-12 | 2.6354 | 1.4082E-12 | 1.9803 | 5.7118E-07 |
| ALPK3 | ENSG00000136383 | 1.3200 | 2.4227E-03 | 1.4544 | 7.8684E-04 | 1.4540 | 1.4144E-03 |
| ALS2CL | ENSG00000178038 | 1.6127 | 6.3622E-08 | 1.1456 | 2.6679E-04 | 1.0342 | 1.9755E-03 |
| AMPD3 | ENSG00000133805 | 1.3280 | 6.9773E-05 | 1.7472 | 1.0507E-07 | 1.6225 | 2.4706E-06 |
| AMY2B | ENSG00000240038 | 2.1035 | 8.2077E-09 | 1.6009 | 3.1132E-05 | 1.1992 | 4.8018E-03 |
| ANKRD24 | ENSG00000089847 | 2.4622 | 1.0797E-12 | 1.8307 | 5.2679E-07 | 1.3383 | 8.9663E-04 |
| ANKRD29 | ENSG00000154065 | 1.9161 | 1.8146E-16 | 1.3444 | 4.8846E-08 | 1.3278 | 2.5293E-07 |
| AOC2 | ENSG00000131480 | 2.1791 | 3.5710E-09 | 1.8017 | 2.7012E-06 | 1.2930 | 2.3803E-03 |
| APOD | ENSG00000189058 | 3.9117 | 1.3099E-06 | 4.2579 | 1.4535E-07 | 2.7451 | 2.8409E-03 |
| ARHGAP27 | ENSG00000159314 | 1.3974 | 1.4271E-04 | 1.3973 | 1.7296E-04 | 1.1591 | 4.0921E-03 |
| ATF3 | ENSG00000162772 | 3.1660 | 4.5504E-13 | 2.5641 | 1.4779E-08 | 2.1540 | 7.7743E-06 |
| ATP6V0D2 | ENSG00000147614 | 4.3384 | 2.6225E-08 | 5.2466 | 1.2855E-11 | 4.1198 | 6.7816E-07 |
| ATP8B4 | ENSG00000104043 | 1.6283 | 5.1015E-08 | 1.4549 | 1.9995E-06 | 1.8375 | 3.2132E-09 |
| AZIN2 | ENSG00000142920 | 1.2861 | 2.6098E-04 | 1.2662 | 3.9767E-04 | 1.0565 | 6.5646E-03 |
| BDKRB2 | ENSG00000168398 | 1.3240 | 9.8752E-06 | 1.3933 | 3.7545E-06 | 1.5747 | 3.8520E-07 |
| BEX4 | ENSG00000102409 | 1.1707 | 1.4519E-08 | 1.2093 | 7.2925E-09 | 1.1095 | 4.3676E-07 |
| BIRC3 | ENSG00000023445 | 2.7307 | 2.3285E-04 | 3.3426 | 4.8685E-06 | 2.7517 | 4.5802E-04 |
| BMP2 | ENSG00000125845 | 3.2858 | 3.1538E-04 | 3.1884 | 5.7336E-04 | 3.1034 | 1.4146E-03 |
| C11orf96 | ENSG00000187479 | 2.7816 | 9.5277E-08 | 3.2109 | 7.4635E-10 | 3.0854 | 1.3217E-08 |
| C1orf226 | ENSG00000239887 | 3.2045 | 6.6303E-10 | 2.2250 | 5.8624E-05 | 1.7005 | 5.4061E-03 |
| C1QTNF1 | ENSG00000173918 | 1.2864 | 1.5505E-09 | 1.5692 | 2.0629E-13 | 1.7463 | 5.6097E-16 |
| C3 | ENSG00000125730 | 2.4427 | 1.2801E-15 | 3.2951 | 5.4343E-27 | 3.7573 | 1.4202E-35 |
| C8orf4 | ENSG00000176907 | 2.5913 | 5.8334E-06 | 3.3776 | 1.3511E-09 | 3.8182 | 1.4621E-11 |
| CA11 | ENSG00000063180 | 1.9601 | 6.0383E-07 | 1.7878 | 9.2989E-06 | 1.2737 | 4.5775E-03 |
| CACNB4 | ENSG00000182389 | 1.7041 | 1.5123E-03 | 1.6991 | 1.7710E-03 | 1.8029 | 1.4224E-03 |
| CAMK1D | ENSG00000183049 | 1.3674 | 1.5286E-09 | 1.2374 | 9.6233E-08 | 1.1155 | 5.2634E-06 |
| CCDC170 | ENSG00000120262 | 2.5078 | 1.5683E-09 | 1.4221 | 2.0920E-03 | 1.3665 | 5.4471E-03 |
| CCDC68 | ENSG00000166510 | 2.6873 | 6.7062E-08 | 3.2343 | 5.9941E-11 | 3.0547 | 3.0066E-09 |
| CCK | ENSG00000187094 | 3.0254 | 1.1836E-09 | 2.6140 | 4.1275E-07 | 2.5473 | 2.5188E-06 |
| CCL2 | ENSG00000108691 | 1.0276 | 1.0895E-03 | 1.5776 | 2.0344E-07 | 1.8051 | 6.4798E-09 |
| CCL20 | ENSG00000115009 | 3.5505 | 7.5257E-03 | 6.4633 | 1.0246E-07 | 6.0984 | 1.6598E-06 |
| CCL5 | ENSG00000271503 | 2.1477 | 4.3200E-03 | 3.6407 | 1.8623E-07 | 3.6234 | 6.2611E-07 |
| CCND2 | ENSG00000118971 | 4.0139 | 5.8102E-06 | 5.0659 | 7.5286E-09 | 2.9997 | 2.0455E-03 |
| CCR7 | ENSG00000126353 | 1.9242 | 7.0778E-06 | 1.7570 | 6.5163E-05 | 1.7878 | 1.0060E-04 |
| CD36 | ENSG00000135218 | 2.1550 | 3.5294E-08 | 2.5195 | 1.1508E-10 | 1.7622 | 3.5367E-05 |
| CD40 | ENSG00000101017 | 1.1174 | 1.3010E-04 | 1.4965 | 1.4312E-07 | 1.2795 | 2.5752E-05 |
| CD82 | ENSG00000085117 | 1.7408 | 1.0535E-09 | 2.0655 | 5.8675E-13 | 1.9659 | 2.8249E-11 |
| CDNF | ENSG00000185267 | 2.4751 | 2.1857E-06 | 2.1487 | 7.5492E-05 | 1.8742 | 1.3681E-03 |
| CELSR3 | ENSG00000008300 | 1.7381 | 1.7794E-17 | 1.2528 | 5.4103E-09 | 1.4247 | 6.8399E-11 |
| CFAP70 | ENSG00000156042 | 2.3009 | 1.5683E-07 | 2.2337 | 5.7543E-07 | 1.4078 | 5.4980E-03 |
| CFH | ENSG00000000971 | 1.0537 | 2.1941E-10 | 1.1469 | 8.1715E-12 | 1.2233 | 1.0324E-12 |
| CHI3L1 | ENSG00000133048 | 2.2451 | 2.1725E-07 | 1.9489 | 1.2800E-05 | 2.3095 | 4.0673E-07 |
| CLCA2 | ENSG00000137975 | 6.8752 | 7.3926E-06 | 7.1744 | 3.2615E-06 | 5.8562 | 4.6744E-04 |
| CMKLR1 | ENSG00000174600 | 2.4247 | 2.2606E-04 | 2.2026 | 1.1374E-03 | 2.0122 | 5.4777E-03 |
| CNNM1 | ENSG00000119946 | 1.1585 | 4.4281E-04 | 1.2803 | 9.9662E-05 | 1.1859 | 6.8019E-04 |
| CNTN4 | ENSG00000144619 | 3.7073 | 2.0461E-07 | 3.5193 | 1.4525E-06 | 3.3678 | 1.2497E-05 |
| CNTNAP2 | ENSG00000174469 | 5.3269 | 6.9302E-04 | 6.6836 | 1.3109E-05 | 4.6637 | 6.6128E-03 |
| CP | ENSG00000047457 | 3.6949 | 7.1563E-06 | 4.3922 | 6.9977E-08 | 4.7947 | 9.9625E-09 |
| CPE | ENSG00000109472 | 1.3096 | 1.4524E-05 | 1.9480 | 2.4549E-11 | 1.1572 | 4.0137E-04 |
| CPT1B | ENSG00000205560 | 1.7141 | 8.3487E-06 | 1.0972 | 8.2053E-03 | 1.5581 | 1.6350E-04 |
| CREBRF | ENSG00000164463 | 1.2804 | 1.5332E-08 | 1.1585 | 5.8240E-07 | 1.0342 | 2.4639E-05 |
| CRELD1 | ENSG00000163703 | 1.0405 | 6.4200E-10 | 1.0865 | 1.8494E-10 | 1.0159 | 1.0978E-08 |
| CST1 | ENSG00000170373 | 3.3625 | 5.7710E-13 | 4.3947 | 6.7946E-21 | 3.2381 | 3.7404E-11 |
| CST2 | ENSG00000170369 | 3.3772 | 9.0072E-09 | 4.1957 | 8.7685E-13 | 3.1593 | 4.1515E-07 |
| CST4 | ENSG00000101441 | 5.4588 | 1.8725E-10 | 6.7130 | 4.8578E-15 | 5.2437 | 7.3030E-09 |
| CTSK | ENSG00000143387 | 1.5616 | 2.1840E-04 | 2.1297 | 2.5934E-07 | 2.1646 | 4.6179E-07 |
| CTSS | ENSG00000163131 | 2.5256 | 1.1187E-05 | 3.6347 | 8.2378E-11 | 3.6760 | 1.5870E-10 |
| CX3CL1 | ENSG00000006210 | 5.4580 | 1.4577E-05 | 4.9761 | 1.2254E-04 | 4.5306 | 1.0580E-03 |
| CXCL1 | ENSG00000163739 | 1.6061 | 6.3437E-04 | 2.4361 | 8.5075E-08 | 3.0093 | 6.2722E-11 |
| CXCL14 | ENSG00000145824 | 2.2180 | 8.1811E-06 | 2.7381 | 2.3071E-08 | 2.1944 | 3.3068E-05 |
| CXCL2 | ENSG00000081041 | 2.0524 | 6.2248E-03 | 2.7345 | 1.6948E-04 | 2.8542 | 1.6201E-04 |
| CXCL5 | ENSG00000163735 | 3.2097 | 2.2192E-06 | 4.7885 | 6.4319E-13 | 4.8067 | 1.9333E-12 |
| CXCL8 | ENSG00000169429 | 3.2144 | 1.2417E-03 | 4.2624 | 1.1936E-05 | 4.1682 | 4.4302E-05 |
| CYP1A1 | ENSG00000140465 | 3.4657 | 1.1504E-05 | 3.4973 | 1.1902E-05 | 2.5897 | 3.1483E-03 |
| CYP26B1 | ENSG00000003137 | 3.4328 | 9.2543E-42 | 3.5386 | 6.2660E-44 | 3.3967 | 3.5468E-40 |
| DAPK2 | ENSG00000035664 | 1.8522 | 4.0338E-05 | 1.9161 | 2.5120E-05 | 1.5675 | 1.5174E-03 |
| DHX58 | ENSG00000108771 | 1.0043 | 1.5647E-03 | 1.1044 | 4.9671E-04 | 1.3003 | 6.3983E-05 |
| DLG2 | ENSG00000150672 | 3.6941 | 5.8875E-04 | 3.9986 | 1.9698E-04 | 3.5511 | 2.0187E-03 |
| DNAH5 | ENSG00000039139 | 1.2216 | 8.0609E-04 | 1.2563 | 6.3588E-04 | 1.3029 | 6.7504E-04 |
| DOCK8 | ENSG00000107099 | 2.1189 | 2.8767E-03 | 3.3463 | 5.9635E-07 | 2.0290 | 7.9899E-03 |
| DTNA | ENSG00000134769 | 2.2645 | 4.7610E-07 | 2.1633 | 2.2683E-06 | 1.9758 | 4.5120E-05 |
| DUSP4 | ENSG00000120875 | 2.6679 | 2.2634E-03 | 3.4639 | 4.7508E-05 | 2.9163 | 1.4076E-03 |
| EEF1A2 | ENSG00000101210 | 1.4502 | 5.8246E-06 | 2.0969 | 2.1603E-11 | 1.6448 | 7.4828E-07 |
| ENPP4 | ENSG00000001561 | 1.1701 | 3.4316E-04 | 1.3224 | 4.9140E-05 | 1.1085 | 1.4883E-03 |
| ENPP5 | ENSG00000112796 | 3.5283 | 7.0090E-14 | 2.9084 | 2.7589E-09 | 2.4743 | 1.9971E-06 |
| F11R | ENSG00000158769 | 1.7658 | 1.3429E-15 | 1.5508 | 8.3754E-12 | 1.2732 | 1.1069E-07 |
| FAM43A | ENSG00000185112 | 1.8583 | 4.4965E-09 | 1.0347 | 2.5393E-03 | 1.0057 | 5.4497E-03 |
| FAM65B | ENSG00000111913 | 1.3848 | 4.5454E-04 | 1.7389 | 7.5395E-06 | 1.9142 | 1.6939E-06 |
| FAM65C | ENSG00000042062 | 2.6987 | 2.6058E-05 | 3.2440 | 3.5149E-07 | 3.4600 | 1.4839E-07 |
| FANK1 | ENSG00000203780 | 1.0488 | 9.2489E-06 | 1.1745 | 6.7421E-07 | 1.1324 | 5.2330E-06 |
| FAXDC2 | ENSG00000170271 | 1.5968 | 7.3524E-12 | 1.1636 | 1.8960E-06 | 1.1327 | 9.9833E-06 |
| FBXL16 | ENSG00000127585 | 2.4646 | 2.1817E-06 | 2.8180 | 5.2335E-08 | 2.0947 | 2.3382E-04 |
| FBXO2 | ENSG00000116661 | 2.4788 | 6.7356E-07 | 2.6661 | 1.0142E-07 | 1.8060 | 1.2307E-03 |
| FBXO32 | ENSG00000156804 | 2.1848 | 1.1607E-09 | 1.6708 | 7.7190E-06 | 1.5400 | 9.3875E-05 |
| FER1L6 | ENSG00000214814 | 4.3040 | 1.8995E-04 | 5.0566 | 8.5041E-06 | 5.1482 | 1.3538E-05 |
| FGD4 | ENSG00000139132 | 1.0725 | 8.7394E-03 | 1.0972 | 7.6610E-03 | 1.2164 | 4.4008E-03 |
| FGF13 | ENSG00000129682 | 2.0158 | 2.6975E-07 | 1.8946 | 2.2344E-06 | 1.8513 | 1.0674E-05 |
| FGL2 | ENSG00000127951 | 1.6939 | 2.3644E-12 | 2.1461 | 1.1612E-18 | 2.0371 | 1.8547E-16 |
| FOSB | ENSG00000125740 | 2.0777 | 3.5255E-03 | 2.6746 | 1.2042E-04 | 2.0630 | 6.4726E-03 |
| FYB | ENSG00000082074 | 5.1703 | 1.5823E-03 | 7.1726 | 4.0728E-06 | 4.8296 | 6.5651E-03 |
| GABARAPL1 | ENSG00000139112 | 1.0573 | 4.3082E-04 | 1.0561 | 5.0564E-04 | 1.0082 | 1.6242E-03 |
| GALNT5 | ENSG00000136542 | 1.1579 | 1.2135E-14 | 1.2635 | 1.1930E-16 | 1.0491 | 3.2562E-11 |
| GCNT3 | ENSG00000140297 | 5.3780 | 1.1054E-03 | 5.3626 | 1.3037E-03 | 5.5263 | 1.4882E-03 |
| GDF15 | ENSG00000130513 | 3.3987 | 1.3349E-04 | 3.7774 | 2.1359E-05 | 3.2346 | 6.3516E-04 |
| GDPD1 | ENSG00000153982 | 1.8767 | 4.9750E-05 | 1.8360 | 9.5090E-05 | 1.4002 | 6.9962E-03 |
| GGT1 | ENSG00000100031 | 1.4154 | 1.9132E-07 | 1.4215 | 2.5700E-07 | 1.8340 | 3.2562E-11 |
| GPC4 | ENSG00000076716 | 1.5142 | 3.7217E-06 | 1.3204 | 8.8049E-05 | 1.2579 | 3.8606E-04 |
| GPNMB | ENSG00000136235 | 1.8178 | 4.7329E-38 | 1.0337 | 2.8918E-12 | 1.1143 | 1.8435E-13 |
| GPRC5C | ENSG00000170412 | 2.4827 | 1.5727E-06 | 2.6306 | 4.3374E-07 | 2.2280 | 6.2068E-05 |
| GRK4 | ENSG00000125388 | 2.4318 | 2.9257E-06 | 1.9854 | 2.8725E-04 | 1.8306 | 1.8085E-03 |
| GS1-259H13.2 | Not mapped | 2.8089 | 8.1852E-06 | 2.8216 | 9.6673E-06 | 1.8985 | 9.2605E-03 |
| HERC5 | ENSG00000138646 | 1.6019 | 2.5811E-03 | 2.1963 | 1.6999E-05 | 2.3036 | 1.4150E-05 |
| HGD | ENSG00000113924 | 2.7724 | 5.8734E-04 | 2.7172 | 9.2311E-04 | 2.6290 | 2.4360E-03 |
| HHAT | ENSG00000054392 | 1.2741 | 2.6421E-04 | 1.6800 | 8.3939E-07 | 1.1464 | 2.3581E-03 |
| HID1 | ENSG00000167861 | 1.6980 | 1.9716E-04 | 2.2948 | 1.9904E-07 | 1.9314 | 4.6119E-05 |
| HIST1H1C | ENSG00000187837 | 3.3302 | 3.0489E-33 | 2.1031 | 3.4391E-13 | 1.6593 | 5.5436E-08 |
| HIST1H2AC | ENSG00000180573 | 2.6031 | 6.4587E-36 | 2.0305 | 6.6972E-21 | 1.6035 | 1.2376E-12 |
| HIST1H2BC | ENSG00000180596 | 2.2429 | 3.8547E-07 | 2.6118 | 2.7253E-09 | 1.7071 | 4.9922E-04 |
| HIST1H2BD | ENSG00000158373 | 2.6672 | 9.2543E-42 | 2.2541 | 1.6870E-28 | 1.9376 | 2.2844E-20 |
| HIST1H2BG | ENSG00000273802 | 2.3970 | 5.4480E-05 | 3.0386 | 1.5491E-07 | 2.1616 | 7.9865E-04 |
| HIST1H2BK | ENSG00000197903 | 1.9528 | 7.4331E-21 | 1.9522 | 5.2973E-20 | 1.4218 | 1.7208E-10 |
| HIST1H3D | ENSG00000197409 | 1.8849 | 5.9427E-05 | 2.7657 | 5.9900E-10 | 1.6900 | 9.5945E-04 |
| HIST1H4H | ENSG00000158406 | 3.2057 | 8.0193E-18 | 2.6916 | 4.0083E-12 | 1.6744 | 1.4559E-04 |
| HIST2H2AA3 | ENSG00000203812 | 2.3257 | 1.3555E-14 | 2.5327 | 1.3372E-16 | 1.8562 | 1.1660E-08 |
| HIST2H2AA4 | ENSG00000272196 | 2.3257 | 1.3555E-14 | 2.5327 | 1.3372E-16 | 1.8562 | 1.1660E-08 |
| HIST2H2BE | ENSG00000184678 | 2.5236 | 3.4736E-21 | 2.5923 | 2.6686E-21 | 1.8900 | 3.3597E-11 |
| HIST2H4A | ENSG00000270882 | 2.9922 | 2.7791E-10 | 2.3761 | 1.8865E-06 | 1.9916 | 2.3715E-04 |
| HIST2H4B | ENSG00000270276 | 2.9922 | 2.7791E-10 | 2.3761 | 1.8865E-06 | 1.9916 | 2.3715E-04 |
| HK2 | ENSG00000159399 | 1.2022 | 2.2453E-03 | 1.2010 | 2.5010E-03 | 1.2487 | 2.5926E-03 |
| HPSE2 | ENSG00000172987 | 5.4137 | 2.8498E-03 | 6.4109 | 3.0602E-04 | 5.4217 | 4.9861E-03 |
| HSD11B1 | ENSG00000117594 | 2.1228 | 3.1125E-08 | 1.9070 | 1.2079E-06 | 1.7585 | 2.1637E-05 |
| HSF4 | ENSG00000102878 | 1.7011 | 1.8414E-08 | 1.0863 | 9.8199E-04 | 1.0250 | 3.5180E-03 |
| IFITM10 | ENSG00000244242 | 1.7748 | 3.9280E-09 | 1.5955 | 2.6877E-07 | 1.1563 | 6.8019E-04 |
| IGFBP2 | ENSG00000115457 | 1.3465 | 4.4315E-03 | 3.2923 | 1.6121E-14 | 2.4407 | 1.0036E-07 |
| IGSF8 | ENSG00000162729 | 1.2545 | 7.8746E-10 | 1.1223 | 8.1488E-08 | 1.0693 | 9.9536E-07 |
| IQCA1 | ENSG00000132321 | 1.4949 | 2.1054E-05 | 1.4381 | 5.9534E-05 | 1.3531 | 3.6871E-04 |
| IQGAP2 | ENSG00000145703 | 3.2712 | 3.3013E-03 | 4.9188 | 2.8088E-06 | 3.8167 | 8.7142E-04 |
| ITGB6 | ENSG00000115221 | 4.7528 | 6.9660E-03 | 6.8251 | 3.7234E-05 | 5.2077 | 4.5613E-03 |
| KCNC3 | ENSG00000131398 | 1.4512 | 2.4788E-03 | 2.4144 | 6.8712E-08 | 2.0875 | 1.2497E-05 |
| KCNH6 | ENSG00000173826 | 3.7843 | 2.1402E-03 | 4.0257 | 1.0550E-03 | 4.2418 | 8.6315E-04 |
| KCNJ2 | ENSG00000123700 | 2.6568 | 1.4593E-11 | 2.0227 | 7.6992E-07 | 2.1224 | 5.4186E-07 |
| KCNJ2-AS1 | ENSG00000267365 | 2.9805 | 4.2885E-09 | 1.9260 | 4.3952E-04 | 1.9650 | 6.0446E-04 |
| KCNJ6 | ENSG00000157542 | 3.7822 | 6.7179E-03 | 4.3369 | 1.5370E-03 | 4.4716 | 1.7560E-03 |
| KCNK1 | ENSG00000135750 | 1.3890 | 1.1158E-05 | 1.5387 | 1.1337E-06 | 1.2642 | 1.9986E-04 |
| KCNK12 | ENSG00000184261 | 1.2187 | 8.9569E-04 | 1.0353 | 6.4260E-03 | 1.2842 | 8.9023E-04 |
| KIAA1211 | ENSG00000109265 | 2.5822 | 9.5380E-13 | 2.0372 | 7.9564E-08 | 1.5760 | 1.3937E-04 |
| KIAA1671 | ENSG00000197077 | 1.5388 | 1.0198E-07 | 1.6852 | 6.3518E-09 | 1.1369 | 3.9544E-04 |
| KLHL24 | ENSG00000114796 | 1.2248 | 2.9516E-05 | 1.1470 | 1.2380E-04 | 1.0331 | 1.1138E-03 |
| KNDC1 | ENSG00000171798 | 2.4355 | 2.2962E-11 | 2.2116 | 3.4027E-09 | 2.0366 | 2.2761E-07 |
| KYNU | ENSG00000115919 | 2.4074 | 3.3137E-07 | 1.9063 | 1.0416E-04 | 1.7767 | 6.2359E-04 |
| L1CAM | ENSG00000198910 | 2.2678 | 1.4368E-07 | 1.8121 | 5.5619E-05 | 1.3427 | 6.6247E-03 |
| LEF1 | ENSG00000138795 | 3.0807 | 3.4512E-03 | 3.5988 | 4.8572E-04 | 3.6500 | 7.1080E-04 |
| LIF | ENSG00000128342 | 1.9901 | 5.7379E-03 | 3.1583 | 4.5097E-06 | 3.0400 | 2.5877E-05 |
| LINC00950 | ENSG00000137103 | 2.6804 | 5.9123E-11 | 1.3574 | 3.1519E-03 | 1.5071 | 1.5050E-03 |
| LINC01021 | ENSG00000250337 | 3.5558 | 9.7697E-05 | 4.3570 | 1.1522E-06 | 2.7981 | 5.9697E-03 |
| LIPG | ENSG00000101670 | 1.6166 | 2.0217E-05 | 1.9601 | 1.8447E-07 | 1.5144 | 1.8394E-04 |
| LOC100129550 | Not mapped | 1.2604 | 4.4380E-10 | 1.4264 | 2.4128E-12 | 1.1349 | 1.4936E-07 |
| LOC100506175 | Not mapped | 3.2558 | 1.2175E-03 | 4.4497 | 2.8193E-06 | 5.1668 | 8.2416E-08 |
| LOC100507156 | Not mapped | 1.1599 | 5.9488E-03 | 1.6284 | 5.9649E-05 | 1.3318 | 2.3694E-03 |
| LOC100630923 | Not mapped | 1.2829 | 9.3250E-09 | 1.0785 | 3.0069E-06 | 1.1071 | 4.2734E-06 |
| LOC115110 | Not mapped | 2.1886 | 6.2945E-05 | 1.8273 | 1.3973E-03 | 1.9618 | 9.4072E-04 |
| LOC344887 | Not mapped | 2.2490 | 1.7438E-12 | 1.7115 | 3.1075E-07 | 1.3747 | 1.4368E-04 |
| LOC728989 | Not mapped | 2.5585 | 2.1786E-03 | 2.3574 | 6.0150E-03 | 2.4730 | 5.8316E-03 |
| LPAR2 | ENSG00000064547 | 1.6593 | 1.6911E-10 | 1.4457 | 7.9237E-08 | 1.2161 | 2.7297E-05 |
| LPAR3 | ENSG00000171517 | 1.9711 | 4.8800E-04 | 2.0119 | 4.2476E-04 | 2.7341 | 1.4339E-06 |
| LPPR3 | ENSG00000282283 | 3.2735 | 3.2842E-06 | 3.1239 | 1.3381E-05 | 2.1274 | 8.6003E-03 |
| LYNX1 | ENSG00000180155 | 1.9883 | 1.6385E-29 | 1.4154 | 1.0555E-14 | 1.2901 | 9.3784E-12 |
| LYPD3 | ENSG00000124466 | 2.0267 | 2.7532E-03 | 2.2733 | 7.1706E-04 | 1.9248 | 8.3132E-03 |
| LYPD5 | ENSG00000159871 | 3.0141 | 6.0364E-08 | 2.6920 | 2.8001E-06 | 1.9205 | 3.0059E-03 |
| MACROD2 | ENSG00000172264 | 3.1870 | 3.3270E-04 | 3.8035 | 1.2344E-05 | 2.8120 | 3.8431E-03 |
| MAP2 | ENSG00000078018 | 2.5879 | 2.4417E-07 | 2.3974 | 3.0840E-06 | 2.0072 | 2.9893E-04 |
| MCTP2 | ENSG00000140563 | 1.2537 | 6.7358E-03 | 1.5282 | 7.9032E-04 | 1.4879 | 1.8852E-03 |
| MGAT3 | ENSG00000128268 | 1.7444 | 3.6602E-03 | 1.6797 | 5.9349E-03 | 1.9598 | 1.7278E-03 |
| MMP12 | ENSG00000262406 | 2.5841 | 5.4121E-06 | 3.1969 | 1.1401E-08 | 2.9165 | 7.4828E-07 |
| MMP3 | ENSG00000149968 | 3.0714 | 5.9148E-04 | 3.3738 | 1.6137E-04 | 3.2189 | 6.2406E-04 |
| MROH7-TTC4 | ENSG00000271723 | 3.9754 | 3.2561E-04 | 3.8194 | 7.0446E-04 | 4.2824 | 2.1649E-04 |
| NAP1L2 | ENSG00000186462 | 1.5150 | 1.0353E-03 | 1.9331 | 1.9212E-05 | 1.3836 | 5.4497E-03 |
| NEB | ENSG00000183091 | 3.1286 | 1.8861E-05 | 3.7280 | 2.5934E-07 | 2.4816 | 2.0612E-03 |
| NEK10 | ENSG00000163491 | 2.0935 | 5.1464E-05 | 2.0754 | 7.3979E-05 | 1.6520 | 3.4698E-03 |
| NFKBIZ | ENSG00000144802 | 1.0542 | 7.5512E-03 | 1.8895 | 3.9491E-07 | 1.7759 | 5.6323E-06 |
| NOTCH3 | ENSG00000074181 | 1.2875 | 9.0198E-09 | 1.2219 | 8.6623E-08 | 1.0193 | 2.6753E-05 |
| NPTX1 | ENSG00000171246 | 2.0447 | 2.5748E-06 | 1.3194 | 4.5030E-03 | 1.2856 | 8.9258E-03 |
| NPTXR | ENSG00000221890 | 2.1264 | 5.7000E-08 | 1.7534 | 1.5034E-05 | 1.2390 | 5.4488E-03 |
| NR4A2 | ENSG00000153234 | 1.7666 | 1.2433E-03 | 2.7532 | 1.6153E-07 | 2.3448 | 2.6595E-05 |
| NR4A3 | ENSG00000119508 | 1.8823 | 9.3534E-08 | 1.9823 | 2.4912E-08 | 1.9194 | 2.4537E-07 |
| NSG1 | ENSG00000168824 | 1.3391 | 5.9610E-03 | 1.4301 | 3.3168E-03 | 1.3601 | 8.4778E-03 |
| NTNG2 | ENSG00000196358 | 1.5647 | 1.5337E-04 | 1.6308 | 8.8051E-05 | 1.4762 | 8.5977E-04 |
| OBSCN | ENSG00000154358 | 1.8418 | 1.6666E-05 | 1.5678 | 3.8426E-04 | 1.5734 | 6.6337E-04 |
| OVGP1 | ENSG00000085465 | 2.2417 | 2.2313E-12 | 1.3684 | 8.0554E-05 | 1.3419 | 2.4066E-04 |
| P2RX6 | ENSG00000099957 | 1.9567 | 6.4006E-04 | 1.9425 | 8.2951E-04 | 1.7871 | 3.8817E-03 |
| P3H2 | ENSG00000090530 | 1.1371 | 1.6894E-07 | 1.3904 | 1.4791E-10 | 1.1242 | 9.3416E-07 |
| PAMR1 | ENSG00000149090 | 1.2309 | 1.7902E-09 | 1.2121 | 5.5504E-09 | 1.2245 | 1.3217E-08 |
| PARD6G | ENSG00000178184 | 2.2825 | 3.5740E-15 | 2.0330 | 1.0637E-11 | 1.5186 | 2.6788E-06 |
| PARK2 | ENSG00000185345 | 3.2340 | 6.2461E-06 | 2.7975 | 1.7253E-04 | 2.5322 | 1.5160E-03 |
| PBXIP1 | ENSG00000163346 | 1.2663 | 2.5991E-12 | 1.3319 | 4.2628E-13 | 1.1273 | 4.4096E-09 |
| PCAT6 | ENSG00000228288 | 1.2911 | 5.5297E-07 | 1.1574 | 1.3369E-05 | 1.2300 | 8.6406E-06 |
| PCDHA4 | ENSG00000204967 | 4.8362 | 3.3815E-05 | 4.5987 | 1.1152E-04 | 4.5108 | 3.0899E-04 |
| PCDHB14 | ENSG00000120327 | 1.6752 | 3.2542E-06 | 1.6437 | 7.0261E-06 | 1.1908 | 3.1904E-03 |
| PCDHB16 | ENSG00000272674 | 1.3159 | 5.0491E-04 | 1.6006 | 1.7047E-05 | 1.3347 | 8.8261E-04 |
| PCDHGB1 | ENSG00000254221 | 1.4009 | 5.1587E-06 | 1.1401 | 3.7873E-04 | 1.2305 | 2.1794E-04 |
| PCSK5 | ENSG00000099139 | 1.5498 | 3.5591E-16 | 1.7702 | 3.7826E-20 | 1.7952 | 2.2844E-20 |
| PCSK6 | ENSG00000140479 | 1.2058 | 2.8767E-03 | 1.3608 | 7.2369E-04 | 1.1365 | 8.8152E-03 |
| PDE4C | ENSG00000105650 | 2.3073 | 2.3448E-09 | 2.2116 | 2.0054E-08 | 2.0521 | 7.0731E-07 |
| PDK4 | ENSG00000004799 | 3.0323 | 2.5215E-06 | 2.7538 | 2.9090E-05 | 2.7784 | 5.4344E-05 |
| PEG3 | ENSG00000198300 | 2.6416 | 6.5991E-07 | 3.1347 | 2.8439E-09 | 1.9056 | 1.2948E-03 |
| PGAM2 | ENSG00000164708 | 1.9815 | 1.9111E-06 | 1.9543 | 3.7545E-06 | 1.5558 | 8.1384E-04 |
| PLA2G4C | ENSG00000105499 | 2.0642 | 5.7486E-10 | 2.1706 | 1.1605E-10 | 1.8297 | 3.2696E-07 |
| PLAT | ENSG00000104368 | 2.1696 | 2.4023E-06 | 2.9868 | 4.7648E-11 | 2.8565 | 1.2375E-09 |
| PLEKHA6 | ENSG00000143850 | 1.9225 | 2.9873E-13 | 1.4949 | 5.0677E-08 | 1.4532 | 3.8777E-07 |
| PLEKHH2 | ENSG00000152527 | 1.0985 | 2.2581E-10 | 1.1014 | 3.9382E-10 | 1.0135 | 3.9534E-08 |
| PPAP2C | ENSG00000141934 | 1.4037 | 6.4425E-05 | 1.6858 | 1.2079E-06 | 1.2851 | 6.7357E-04 |
| PPFIA3 | ENSG00000177380 | 1.2164 | 2.6889E-03 | 1.4315 | 3.3513E-04 | 1.3491 | 1.4577E-03 |
| PROS1 | ENSG00000184500 | 1.1588 | 6.2255E-05 | 1.0172 | 6.3704E-04 | 1.0213 | 1.0580E-03 |
| PTCHD4 | ENSG00000244694 | 4.8875 | 4.1922E-16 | 4.4756 | 4.2628E-13 | 3.5134 | 1.0036E-07 |
| QPRT | ENSG00000103485 | 2.8438 | 2.1250E-08 | 2.7664 | 8.6623E-08 | 2.7189 | 4.6179E-07 |
| RAB27B | ENSG00000041353 | 3.3913 | 2.3926E-08 | 3.0639 | 9.2179E-07 | 2.5366 | 1.5924E-04 |
| RAB43 | ENSG00000172780 | 1.1970 | 1.3011E-03 | 1.5661 | 1.6990E-05 | 1.3193 | 6.9119E-04 |
| RAP1GAP | ENSG00000076864 | 2.7332 | 3.7060E-07 | 2.5133 | 5.1311E-06 | 2.1454 | 2.9850E-04 |
| RASL10B | ENSG00000270885 | 1.4627 | 2.6899E-06 | 1.1027 | 8.5505E-04 | 1.2467 | 2.4688E-04 |
| RASL12 | ENSG00000103710 | 1.9124 | 1.1677E-03 | 2.2492 | 1.0660E-04 | 1.9764 | 1.5079E-03 |
| RELB | ENSG00000104856 | 1.3953 | 6.8018E-07 | 1.8886 | 7.6826E-12 | 1.6076 | 3.6269E-08 |
| RENBP | ENSG00000102032 | 2.4461 | 1.3142E-05 | 2.3479 | 4.1674E-05 | 1.7338 | 6.8367E-03 |
| RFTN2 | ENSG00000162944 | 2.5490 | 3.5525E-09 | 3.0790 | 8.3253E-13 | 2.9632 | 2.6027E-11 |
| RGS11 | ENSG00000076344 | 1.9017 | 9.5116E-08 | 1.1150 | 3.9101E-03 | 1.1289 | 5.4777E-03 |
| RND3 | ENSG00000115963 | 1.0334 | 4.1166E-05 | 1.0352 | 4.8704E-05 | 1.0622 | 6.4037E-05 |
| ROBO2 | ENSG00000185008 | 1.2637 | 3.0197E-06 | 1.6666 | 3.5288E-10 | 1.1698 | 5.9174E-05 |
| RRAD | ENSG00000166592 | 3.4489 | 7.9520E-16 | 2.2151 | 9.8462E-07 | 1.7768 | 2.6039E-04 |
| RRAGD | ENSG00000025039 | 3.5548 | 7.9410E-08 | 3.0381 | 9.4178E-06 | 2.7214 | 1.9518E-04 |
| RYR2 | ENSG00000198626 | 4.3214 | 1.0927E-03 | 4.3294 | 1.2084E-03 | 4.0940 | 3.9845E-03 |
| SAT1 | ENSG00000130066 | 1.3273 | 2.0375E-08 | 1.7807 | 4.2326E-14 | 1.3354 | 8.5827E-08 |
| SCN4B | ENSG00000177098 | 2.5554 | 4.6050E-06 | 2.7201 | 1.2286E-06 | 2.1135 | 4.7848E-04 |
| SCN5A | ENSG00000183873 | 1.4205 | 1.6900E-05 | 1.6169 | 8.7990E-07 | 1.6481 | 1.4514E-06 |
| SCUBE1 | ENSG00000159307 | 2.5835 | 1.8713E-05 | 2.0390 | 1.4035E-03 | 2.7913 | 9.8901E-06 |
| SDK1 | ENSG00000146555 | 1.8666 | 3.5951E-12 | 1.4941 | 9.5480E-08 | 1.7663 | 5.1580E-10 |
| SDK2 | ENSG00000069188 | 2.8015 | 5.3789E-04 | 3.7983 | 1.2892E-06 | 3.2846 | 8.7397E-05 |
| SEPP1 | ENSG00000250722 | 1.5626 | 2.0009E-04 | 1.2027 | 6.5448E-03 | 1.5140 | 7.2343E-04 |
| SERPINI1 | ENSG00000163536 | 1.7589 | 5.7112E-09 | 2.0954 | 3.1167E-12 | 1.5468 | 2.2233E-06 |
| SESN3 | ENSG00000149212 | 1.8201 | 6.6166E-15 | 1.7433 | 3.2073E-13 | 1.2727 | 6.7112E-07 |
| SEZ6L2 | ENSG00000174938 | 1.7501 | 3.6195E-22 | 1.6076 | 4.6821E-18 | 1.5991 | 1.9291E-17 |
| SGIP1 | ENSG00000118473 | 1.6057 | 1.4287E-07 | 1.7042 | 3.0334E-08 | 1.4742 | 5.9164E-06 |
| SH3BGRL2 | ENSG00000198478 | 1.7857 | 3.0167E-15 | 1.6220 | 3.5914E-12 | 1.6361 | 9.5124E-12 |
| SHANK1 | ENSG00000161681 | 1.5608 | 1.1733E-05 | 1.2153 | 1.1468E-03 | 1.3120 | 7.1041E-04 |
| SHC2 | ENSG00000129946 | 4.0633 | 9.3250E-09 | 4.3584 | 9.5330E-10 | 3.9492 | 1.4355E-07 |
| SHC4 | ENSG00000185634 | 1.6867 | 6.3492E-08 | 1.9330 | 6.2588E-10 | 1.7948 | 3.9989E-08 |
| SLC16A6 | ENSG00000108932 | 2.8676 | 4.5972E-03 | 3.3423 | 7.9171E-04 | 3.1291 | 3.0898E-03 |
| SLC1A2 | ENSG00000110436 | 1.2998 | 1.1732E-03 | 1.4594 | 2.5035E-04 | 1.3711 | 1.1450E-03 |
| SLC2A12 | ENSG00000146411 | 1.7763 | 4.1442E-07 | 1.3341 | 2.7877E-04 | 1.2130 | 1.8693E-03 |
| SLC44A3 | ENSG00000143036 | 1.4671 | 2.8640E-05 | 1.2115 | 9.4244E-04 | 1.0514 | 7.9975E-03 |
| SLC4A7 | ENSG00000033867 | 1.3703 | 4.6049E-04 | 1.3382 | 7.4630E-04 | 1.1637 | 6.0782E-03 |
| SLC6A12 | ENSG00000111181 | 4.3445 | 1.6875E-04 | 4.1860 | 3.8134E-04 | 3.9477 | 1.6513E-03 |
| SLC6A15 | ENSG00000072041 | 3.2520 | 4.8800E-04 | 3.5598 | 1.3300E-04 | 2.8451 | 4.9821E-03 |
| SLC7A8 | ENSG00000092068 | 1.8529 | 3.8688E-03 | 2.5218 | 4.7897E-05 | 2.2275 | 7.4557E-04 |
| SLCO2B1 | ENSG00000137491 | 3.1861 | 1.2087E-03 | 4.7030 | 4.2369E-07 | 3.5481 | 5.1599E-04 |
| SMIM10L2B | ENSG00000196972 | 2.0449 | 6.2904E-09 | 1.6400 | 9.7369E-06 | 1.6261 | 3.0940E-05 |
| SNCA | ENSG00000145335 | 2.9459 | 4.1807E-05 | 3.6708 | 1.7044E-07 | 3.1591 | 2.7415E-05 |
| SOD2 | ENSG00000112096 | 1.8311 | 2.3579E-06 | 1.7313 | 1.1650E-05 | 1.7452 | 2.2869E-05 |
| SPINT1 | ENSG00000166145 | 2.2172 | 1.6525E-08 | 2.4889 | 2.6145E-10 | 1.7814 | 3.3802E-05 |
| SPRY1 | ENSG00000164056 | 1.3186 | 6.4299E-03 | 1.8378 | 8.6074E-05 | 1.6666 | 7.7293E-04 |
| SQSTM1 | ENSG00000161011 | 1.6306 | 1.6907E-13 | 1.5799 | 2.6180E-12 | 1.3657 | 7.0795E-09 |
| SRGAP3 | ENSG00000196220 | 3.3822 | 7.3305E-18 | 2.3894 | 9.1090E-09 | 1.6036 | 5.7477E-04 |
| SSPO | ENSG00000197558 | 3.1204 | 2.9255E-13 | 2.2342 | 9.2148E-07 | 1.9784 | 5.0155E-05 |
| STAT1 | ENSG00000115415 | 1.0324 | 3.2962E-05 | 1.2660 | 2.8044E-07 | 1.3396 | 1.4876E-07 |
| STC1 | ENSG00000159167 | 2.8093 | 3.7705E-03 | 3.3431 | 4.8572E-04 | 3.1933 | 1.5511E-03 |
| STOM | ENSG00000148175 | 1.2454 | 5.1563E-05 | 1.1517 | 2.4258E-04 | 1.2711 | 8.9830E-05 |
| SVIL | ENSG00000197321 | 1.6760 | 7.5821E-04 | 1.9603 | 7.3135E-05 | 1.8102 | 5.1599E-04 |
| SYNGR1 | ENSG00000100321 | 1.3599 | 3.6529E-11 | 1.0232 | 1.8003E-06 | 1.2171 | 2.8448E-08 |
| SYNGR3 | ENSG00000127561 | 1.1614 | 7.2440E-08 | 1.2077 | 3.0334E-08 | 1.5205 | 3.7659E-12 |
| TAC3 | ENSG00000166863 | 5.3068 | 8.0380E-08 | 5.0396 | 6.3787E-07 | 5.0092 | 2.1025E-06 |
| TANC1 | ENSG00000115183 | 1.0608 | 1.6864E-05 | 1.0749 | 1.5734E-05 | 1.0888 | 2.8260E-05 |
| TARID | ENSG00000227954 | 3.0069 | 6.9718E-12 | 3.4963 | 2.2599E-15 | 3.2443 | 1.0324E-12 |
| TENM1 | ENSG00000009694 | 2.6538 | 1.2679E-07 | 2.3416 | 5.9132E-06 | 1.9973 | 3.1881E-04 |
| TG | ENSG00000042832 | 2.8261 | 2.1792E-13 | 3.0995 | 1.7818E-15 | 1.9514 | 7.0122E-06 |
| TLL2 | ENSG00000095587 | 1.9425 | 1.2409E-04 | 3.1316 | 4.9695E-11 | 1.6330 | 3.4932E-03 |
| TMEM132A | ENSG00000006118 | 1.3470 | 1.1270E-17 | 1.3290 | 1.3372E-16 | 1.4528 | 2.7470E-19 |
| TMEM140 | ENSG00000146859 | 1.2093 | 4.7171E-15 | 1.3706 | 1.8638E-18 | 1.4049 | 5.0632E-19 |
| TMEM150C | ENSG00000249242 | 2.1223 | 1.0536E-03 | 2.1780 | 8.6109E-04 | 2.2657 | 8.6831E-04 |
| TMEM178A | ENSG00000152154 | 1.9653 | 1.3043E-07 | 2.1385 | 1.1034E-08 | 2.1615 | 2.6824E-08 |
| TMEM217 | ENSG00000172738 | 1.2448 | 9.6072E-07 | 1.7674 | 9.3368E-13 | 1.3395 | 4.9135E-07 |
| TMEM229B | ENSG00000198133 | 2.0779 | 2.5468E-05 | 2.1855 | 1.0288E-05 | 1.7571 | 1.1490E-03 |
| TMEM38A | ENSG00000072954 | 1.6037 | 7.1369E-05 | 1.2835 | 2.5400E-03 | 1.2655 | 4.9605E-03 |
| TMEM56 | Not mapped | 1.5929 | 3.0634E-03 | 1.5696 | 3.9860E-03 | 1.6371 | 4.0950E-03 |
| TMEM59L | ENSG00000105696 | 1.3646 | 4.4784E-07 | 1.1814 | 2.1184E-05 | 1.2747 | 9.5433E-06 |
| TMEM74B | ENSG00000125895 | 2.2462 | 3.5413E-03 | 2.4477 | 1.3883E-03 | 2.4548 | 2.2845E-03 |
| TMOD1 | ENSG00000136842 | 2.3416 | 5.3649E-16 | 2.3395 | 2.2148E-15 | 1.9640 | 1.5870E-10 |
| TNFRSF10C | ENSG00000173535 | 2.3545 | 6.0644E-22 | 2.1687 | 5.9230E-18 | 1.3723 | 5.0389E-07 |
| TNFSF13B | ENSG00000102524 | 1.4583 | 7.1983E-05 | 1.6292 | 8.5186E-06 | 1.9110 | 3.0492E-07 |
| TPCN1 | ENSG00000186815 | 1.3885 | 2.9824E-13 | 1.2926 | 3.1787E-11 | 1.1055 | 6.8664E-08 |
| TRABD2B | ENSG00000269113 | 1.0391 | 1.4246E-03 | 1.2487 | 1.0260E-04 | 1.3680 | 3.9143E-05 |
| TRIM74 | ENSG00000155428 | 3.1649 | 7.6303E-06 | 2.3173 | 2.2164E-03 | 2.3117 | 3.7738E-03 |
| TSC22D1-AS1 | ENSG00000278156 | 1.4746 | 3.2320E-04 | 1.4928 | 3.1453E-04 | 1.3127 | 3.3363E-03 |
| TSHZ2 | ENSG00000182463 | 1.5333 | 3.6715E-05 | 1.5974 | 1.9393E-05 | 1.5269 | 1.0870E-04 |
| TSLP | ENSG00000145777 | 1.8504 | 3.3778E-04 | 2.0954 | 4.6530E-05 | 1.7245 | 1.7951E-03 |
| TSPAN11 | ENSG00000110900 | 3.7234 | 5.8149E-07 | 3.6281 | 1.5964E-06 | 2.8552 | 4.5710E-04 |
| UBE2QL1 | ENSG00000215218 | 3.4722 | 9.7114E-03 | 4.4976 | 4.2476E-04 | 4.2389 | 1.8152E-03 |
| VCAM1 | ENSG00000162692 | 2.4806 | 7.3814E-10 | 2.5971 | 1.9092E-10 | 2.7854 | 2.5990E-11 |
| WFS1 | ENSG00000109501 | 1.1781 | 9.6682E-06 | 1.1924 | 9.3662E-06 | 1.1176 | 7.9273E-05 |
| XXYLT1-AS2 | ENSG00000230266 | 2.3436 | 7.6445E-05 | 1.9000 | 2.2784E-03 | 2.1158 | 9.6617E-04 |
| YPEL2 | ENSG00000175155 | 1.3278 | 2.6282E-06 | 1.5367 | 5.2159E-08 | 1.2722 | 2.3525E-05 |
| ZMIZ1-AS1 | ENSG00000224596 | 2.2853 | 2.8837E-04 | 2.2592 | 4.0694E-04 | 1.9477 | 4.7007E-03 |
| ZNF582-AS1 | Not mapped | 1.3162 | 2.7350E-05 | 1.2254 | 1.3720E-04 | 1.2185 | 3.0879E-04 |
| ZNF610 | ENSG00000167554 | 1.3828 | 2.7648E-06 | 1.4412 | 1.2702E-06 | 1.2344 | 1.0689E-04 |
| ZNF667 | ENSG00000198046 | 1.7997 | 1.3457E-09 | 1.7661 | 5.0038E-09 | 1.6014 | 4.6179E-07 |
| ZNF702P | ENSG00000242779 | 1.7397 | 2.4470E-12 | 1.7296 | 8.3754E-12 | 1.3253 | 1.2532E-06 |
| ZSCAN18 | ENSG00000121413 | 1.4122 | 3.2739E-17 | 1.3341 | 7.5239E-15 | 1.0723 | 2.8576E-09 |
