# Supplementary material for: Mapping the core senescence phenotype of primary human colon fibroblasts
Source: Aging (Albany NY). 2024 Feb 21;16(4):3068–87. doi: 10.18632/aging.205577 (PMC10929841; doi:10.18632/aging.205577)
Supplement: Supplementary Table 4A [file aging-16-205577-s004.docx]

**Supplemental Table 4A. Gene ontology analysis for biological processes on the core senescence profile (FDR ≤ 0.01).**

| **GO_ID** | **Pathway** | **Fold enrichment** | **Enrichment FDR** | **N genes** | **Pathway genes** | **Gene ratio** | **genes** |
| --- | --- | --- | --- | --- | --- | --- | --- |
| GO:1990868 | Response to chemokine | 11.150 | 1.10E-06 | 12 | 114 | 10.53 | CX3CL1 CXCL2 DOCK8 CCL2 CCL20 CCR7 ACKR3 CXCL5 CXCL1 CXCL8 CMKLR1 CCL5 |
| GO:0007186 | G protein-coupled receptor signaling pathway | 3.231 | 5.06E-06 | 32 | 1569 | 2.04 | CX3CL1 CELSR3 TENM1 LPAR2 RGS11 CXCL2 PDE4C CCL2 CPE CCL20 ADGRB2 GRK4 C3 CCR7 FGD4 ACKR3 SNCA IQGAP2 OBSCN ADCY8 CXCL5 CXCL1 TAC3 BDKRB2 CXCL8 GPRC5C LPAR3 CMKLR1 ADGRB1 CCK CCL5 ADGRL1 |
| GO:0006954 | Inflammatory response | 3.088 | 7.51E-06 | 32 | 950 | 3.37 | CYP26B1 CX3CL1 BIRC3 CXCL2 GGT1 CD40 RELB PLA2G4C CCL2 CCL20 ITGB6 C3 BMP2 CCR7 CHI3L1 CD36 NFKBIZ SNCA TSLP MMP3 ADCY8 F11R SCUBE1 VCAM1 CXCL5 CXCL1 BDKRB2 CXCL8 CMKLR1 CAMK1D APOD CCL5 |
| GO:0009617 | Response to bacterium | 3.644 | 7.51E-06 | 26 | 741 | 3.51 | CX3CL1 CXCL2 GGT1 CD40 CCL2 DHX58 SOD2 CCL20 C3 BMP2 CCR7 CD36 CYP1A1 SNCA TSLP VCAM1 CXCL5 CXCL1 CXCL8 MACROD2 H2BC4 ADGRB1 H2BC12 TMEM229B FER1L6 CCL5 |
| GO:0061844 | Antimicrobial humoral immune response mediated by antimicrobial peptide | 16.726 | 7.51E-06 | 8 | 92 | 8.70 | CXCL2 TSLP CXCL14 CXCL5 CXCL1 CXCL8 H2BC4 H2BC12 |
| GO:0006811 | Ion transport | 2.296 | 2.51E-05 | 47 | 1720 | 2.73 | CX3CL1 SLC4A7 TG CP SLC6A15 TMEM38A SLC7A8 P2RX6 CCL2 SLC16A6 WFS1 SLC1A2 SLC6A12 KLHL24 CNNM1 KCNJ2 ABCC4 CCR7 FGF13 KCNC3 CD36 KCNK1 SLCO2B1 CLCA2 SNCA ATP6V0D2 STOM DLG2 AKAP6 KCNJ6 STC1 SHANK1 CTSS RRAD BDKRB2 LPAR3 KCNH6 SCN4B SCN5A KCNK12 PRKN TPCN1 CCK RYR2 TMEM150C CCL5 |
| GO:0007155 | Cell adhesion | 2.240 | 4.09E-05 | 47 | 1639 | 2.87 | CX3CL1 CELSR3 TENM1 SDK2 GPC4 TNFSF13B DOCK8 CCL2 ITGB6 IGFBP2 RND3 NR4A3 PCDHB14 LYPD3 BMP2 CCR7 FGL2 LIF CD36 GPNMB CLCA2 LEF1 ACKR3 CNTN4 NFKBIZ SDK1 DLG2 OBSCN F11R LYPD5 VCAM1 SERPINI1 CXCL8 C1QTNF1 ACER2 ADGRB1 ROBO2 APOD NTNG2 L1CAM PCDHA4 PCDHGB1 MMP12 PCDHB16 CCL5 CNTNAP2 ADGRL1 |
| GO:0006959 | Humoral immune response | 6.154 | 4.63E-05 | 13 | 331 | 3.93 | CFH CXCL2 CCL2 C3 CCR7 TSLP CXCL14 CXCL5 CXCL1 CXCL8 H2BC4 PROS1 H2BC12 |
| GO:0006812 | Cation transport | 2.389 | 1.53E-04 | 37 | 1286 | 2.88 | CX3CL1 SLC4A7 CP SLC6A15 TMEM38A SLC7A8 P2RX6 CCL2 WFS1 SLC1A2 SLC6A12 KLHL24 KCNJ2 CCR7 FGF13 KCNC3 KCNK1 SNCA ATP6V0D2 STOM DLG2 AKAP6 KCNJ6 STC1 SHANK1 CTSS RRAD LPAR3 KCNH6 SCN4B SCN5A KCNK12 PRKN TPCN1 RYR2 CCL5 |
| GO:0055085 | Transmembrane transport | 2.116 | 1.80E-04 | 46 | 1764 | 2.61 | CX3CL1 SLC4A7 SLC6A15 TMEM38A SLC7A8 P2RX6 CCL2 SLC16A6 SLC1A2 SLC6A12 KLHL24 NR4A3 CNNM1 KCNJ2 ABCC4 C3 CCR7 FGF13 KCNC3 CD36 KCNK1 SLCO2B1 CLCA2 AZIN2 SLC44A3 SNCA SLC2A12 ATP6V0D2 STOM DLG2 AKAP6 ABCA9 KCNJ6 HK2 SHANK1 CTSS RRAD KCNH6 SCN4B SCN5A KCNK12 TPCN1 RYR2 CPT1B TMEM150C |
| GO:0007267 | Cell-cell signaling | 2.022 | 2.46E-04 | 49 | 1885 | 2.60 | CX3CL1 CELSR3 GPC4 PCSK5 P2RX6 SYNGR1 PLAT CCL2 CPE SLC1A2 CCL20 IGFBP2 PCDHB14 ABCC4 C3 BMP2 LIF FGF13 GDF15 DTNA GPNMB LEF1 MCTP2 CNTN4 SNCA CXCL14 DLG2 NR4A2 ADCY8 SQSTM1 SHANK1 CXCL5 NSG1 NPTX1 LPAR3 C1QTNF1 SCN4B PPFIA3 LYNX1 ADGRB1 SCN5A PRKN NTNG2 RYR2 NPTXR TRABD2B RASL10B PCDHB16 CCL5 |
| GO:0050801 | Ion homeostasis | 2.655 | 2.80E-04 | 29 | 839 | 3.46 | PDK4 CX3CL1 SLC4A7 CP LPAR2 TMEM38A SLC7A8 CD40 WFS1 KCNJ2 CCR7 CD36 ACKR3 SNCA ATP6V0D2 AKAP6 TMEM178A ADCY8 STC1 BDKRB2 LPAR3 C1QTNF1 CMKLR1 SCN5A PRKN TPCN1 CCK RYR2 CCL5 |
| GO:0072507 | Divalent inorganic cation homeostasis | 3.272 | 2.95E-04 | 21 | 531 | 3.95 | CX3CL1 LPAR2 TMEM38A CD40 WFS1 CCR7 CD36 ACKR3 SNCA AKAP6 TMEM178A ADCY8 STC1 BDKRB2 LPAR3 C1QTNF1 CMKLR1 PRKN TPCN1 RYR2 CCL5 |
| GO:0060326 | Cell chemotaxis | 4.096 | 2.95E-04 | 16 | 343 | 4.66 | CX3CL1 DAPK2 CXCL2 CCL2 CCL20 CCR7 LEF1 ACKR3 CXCL14 VCAM1 CXCL5 CXCL1 CXCL8 CMKLR1 CAMK1D CCL5 |
| GO:0051050 | Positive regulation of transport | 2.386 | 3.04E-04 | 34 | 1013 | 3.36 | CX3CL1 TENM1 RAB27B MAP2 LIPG CCL2 WFS1 SLC1A2 SGIP1 NR4A3 KCNJ2 C3 BMP2 SYNGR3 CD36 AZIN2 SNCA STOM AKAP6 ADCY8 KCNJ6 STC1 CTSS CREBRF LPAR3 C1QTNF1 SCN4B CAMK1D SCN5A PRKN CCK RYR2 RASL10B CCL5 |
| GO:0019730 | Antimicrobial humoral response | 9.123 | 3.04E-04 | 8 | 166 | 4.82 | CXCL2 TSLP CXCL14 CXCL5 CXCL1 CXCL8 H2BC4 H2BC12 |
| GO:0030001 | Metal ion transport | 2.505 | 4.42E-04 | 30 | 984 | 3.05 | CX3CL1 SLC4A7 CP SLC6A15 TMEM38A CCL2 WFS1 SLC6A12 KLHL24 KCNJ2 CCR7 FGF13 KCNC3 KCNK1 SNCA ATP6V0D2 STOM AKAP6 KCNJ6 STC1 RRAD LPAR3 KCNH6 SCN4B SCN5A KCNK12 TPCN1 RYR2 CCL5 |
| GO:0048878 | Chemical homeostasis | 2.226 | 4.58E-04 | 37 | 1264 | 2.93 | CYP26B1 PDK4 CX3CL1 SLC4A7 CP LPAR2 TMEM38A AKR1B1 SLC7A8 CD40 LIPG WFS1 SOD2 ITGB6 STAT1 KCNJ2 CCR7 CD36 ACKR3 SNCA ATP6V0D2 SESN3 AKAP6 TMEM178A ADCY8 STC1 HK2 BDKRB2 LPAR3 C1QTNF1 CMKLR1 SCN5A PRKN TPCN1 CCK RYR2 CCL5 |
| GO:0051480 | Regulation of cytosolic calcium ion concentration | 3.752 | 6.67E-04 | 16 | 361 | 4.43 | CX3CL1 LPAR2 TMEM38A CCR7 CD36 ACKR3 SNCA AKAP6 TMEM178A ADCY8 BDKRB2 LPAR3 C1QTNF1 CMKLR1 TPCN1 RYR2 |
| GO:0006576 | Cellular biogenic amine metabolic process | 6.641 | 8.03E-04 | 9 | 110 | 8.18 | AKR1B1 KYNU SAT1 AOC2 AZIN2 SNCA NR4A2 GDPD1 PRKN |
| GO:0007204 | Positive regulation of cytosolic calcium ion concentration | 3.880 | 8.11E-04 | 15 | 325 | 4.62 | CX3CL1 LPAR2 TMEM38A CCR7 CD36 ACKR3 SNCA AKAP6 ADCY8 BDKRB2 LPAR3 C1QTNF1 CMKLR1 TPCN1 RYR2 |
| GO:0034220 | Ion transmembrane transport | 2.233 | 8.79E-04 | 34 | 1251 | 2.72 | CX3CL1 SLC4A7 SLC6A15 TMEM38A SLC7A8 P2RX6 CCL2 SLC1A2 SLC6A12 KLHL24 KCNJ2 ABCC4 CCR7 FGF13 KCNC3 KCNK1 CLCA2 SNCA ATP6V0D2 STOM DLG2 AKAP6 KCNJ6 SHANK1 CTSS RRAD KCNH6 SCN4B SCN5A KCNK12 TPCN1 RYR2 TMEM150C |
| GO:0043270 | Positive regulation of ion transport | 3.840 | 8.79E-04 | 15 | 318 | 4.72 | CX3CL1 CCL2 WFS1 KCNJ2 SNCA AKAP6 KCNJ6 STC1 CTSS LPAR3 SCN4B SCN5A CCK RYR2 CCL5 |
| GO:0030534 | Adult behavior | 5.575 | 1.04E-03 | 10 | 138 | 7.25 | SLC1A2 CCND2 TMOD1 SNCA SDK1 NR4A2 SHANK1 SEZ6L2 PRKN CNTNAP2 |
| GO:0010959 | Regulation of metal ion transport | 3.169 | 1.38E-03 | 18 | 448 | 4.02 | CX3CL1 TMEM38A CCL2 WFS1 KLHL24 KCNJ2 FGF13 SNCA STOM AKAP6 KCNJ6 STC1 RRAD LPAR3 SCN4B SCN5A RYR2 CCL5 |
| GO:0019722 | Calcium-mediated signaling | 4.427 | 1.42E-03 | 12 | 218 | 5.50 | TMEM38A CCL20 ADGRB2 CCR7 MCTP2 ACKR3 AKAP6 VCAM1 CXCL8 CMKLR1 RYR2 ADGRL1 |
| GO:0051049 | Regulation of transport | 1.841 | 1.64E-03 | 48 | 1910 | 2.51 | CX3CL1 TENM1 RAB27B TMEM38A MAP2 LIPG CCL2 WFS1 SLC1A2 KLHL24 SGIP1 NR4A3 KCNJ2 C3 BMP2 SYNGR3 LIF FGF13 KCNC3 CD36 MCTP2 AZIN2 SNCA STOM DLG2 AKAP6 ADCY8 KCNJ6 STC1 HK2 SHANK1 CTSS CREBRF RRAD LPAR3 KCNH6 C1QTNF1 SCN4B CAMK1D SCN5A KCNK12 PRKN TPCN1 CCK APOD RYR2 RASL10B CCL5 |
| GO:0040011 | Locomotion | 1.819 | 1.80E-03 | 49 | 1982 | 2.47 | CX3CL1 DAPK2 DNAH5 NOTCH3 GPC4 RAP1GAP CXCL2 SLC7A8 CD40 BEX4 PLAT DOCK8 CCL2 TSPAN11 SOD2 CCL20 ITGB6 RND3 NR4A3 BMP2 CCR7 MGAT3 FGF13 GPNMB LEF1 ACKR3 CNTN4 SNCA CXCL14 NR4A2 F11R STC1 VCAM1 IGSF8 CXCL5 CXCL1 CXCL8 CMKLR1 ADGRB1 CAMK1D PROS1 ROBO2 CCK APOD SRGAP3 NTNG2 L1CAM MMP12 CCL5 |
| GO:0099536 | Synaptic signaling | 2.489 | 1.88E-03 | 25 | 815 | 3.07 | CX3CL1 P2RX6 SYNGR1 PLAT CCL2 SLC1A2 PCDHB14 DTNA MCTP2 CNTN4 SNCA DLG2 ADCY8 SQSTM1 SHANK1 NSG1 NPTX1 LPAR3 PPFIA3 LYNX1 ADGRB1 PRKN NTNG2 NPTXR PCDHB16 |
| GO:0070371 | ERK1 and ERK2 cascade | 3.501 | 1.91E-03 | 15 | 350 | 4.29 | CX3CL1 CCL2 CCL20 DUSP4 BMP2 CCR7 LIF CHI3L1 CD36 GPNMB ACKR3 ATF3 NEK10 SPRY1 CCL5 |
| GO:0009308 | Amine metabolic process | 4.451 | 2.48E-03 | 11 | 191 | 5.76 | AKR1B1 KYNU SAT1 AOC2 CYP1A1 AZIN2 SNCA NR4A2 GDPD1 VCAM1 PRKN |
| GO:0050909 | Sensory perception of taste | 12.544 | 2.48E-03 | 5 | 97 | 5.15 | CST4 CD36 LEF1 CST2 CST1 |
| GO:0016477 | Cell migration | 1.900 | 2.49E-03 | 42 | 1590 | 2.64 | CX3CL1 DAPK2 GPC4 CXCL2 SLC7A8 CD40 BEX4 PLAT DOCK8 CCL2 TSPAN11 SOD2 CCL20 ITGB6 RND3 NR4A3 BMP2 CCR7 MGAT3 FGF13 GPNMB LEF1 ACKR3 CXCL14 NR4A2 F11R STC1 VCAM1 CXCL5 CXCL1 CXCL8 CMKLR1 ADGRB1 CAMK1D PROS1 CCK APOD SRGAP3 NTNG2 L1CAM MMP12 CCL5 |
| GO:0032496 | Response to lipopolysaccharide | 3.360 | 2.81E-03 | 15 | 381 | 3.94 | CX3CL1 CXCL2 GGT1 CD40 CCL2 SOD2 CCR7 CD36 CYP1A1 SNCA VCAM1 CXCL5 CXCL1 CXCL8 CCL5 |
| GO:0009607 | Response to biotic stimulus | 1.960 | 3.05E-03 | 38 | 1905 | 1.99 | CFH CX3CL1 BIRC3 CXCL2 GGT1 CD40 RELB CCL2 DHX58 WFS1 SOD2 CCL20 ITGB6 STAT1 KYNU C3 BMP2 CCR7 FGL2 CD36 HERC5 CYP1A1 SNCA TSLP CXCL14 VCAM1 CXCL5 CXCL1 CXCL8 MACROD2 RAB43 H2BC4 ADGRB1 H2BC12 TMEM229B FER1L6 MMP12 CCL5 |
| GO:0051707 | Response to other organism | 1.975 | 3.20E-03 | 37 | 1866 | 1.98 | CFH CX3CL1 BIRC3 CXCL2 GGT1 CD40 RELB CCL2 DHX58 SOD2 CCL20 ITGB6 STAT1 KYNU C3 BMP2 CCR7 FGL2 CD36 HERC5 CYP1A1 SNCA TSLP CXCL14 VCAM1 CXCL5 CXCL1 CXCL8 MACROD2 RAB43 H2BC4 ADGRB1 H2BC12 TMEM229B FER1L6 MMP12 CCL5 |
| GO:0009991 | Response to extracellular stimulus | 2.621 | 3.40E-03 | 21 | 561 | 3.74 | CYP26B1 PDK4 RRAGD CD40 LIPG SLC1A2 SOD2 STAT1 IGFBP2 KYNU SGIP1 GDF15 GABARAPL1 CYP1A1 ATP6V0D2 SESN3 NR4A2 STC1 VCAM1 ATF3 CCK |
| GO:0007416 | Synapse assembly | 4.213 | 3.67E-03 | 11 | 186 | 5.91 | SDK2 GPC4 PCDHB14 FGF13 SNCA SDK1 SHANK1 ADGRB1 ROBO2 NTNG2 PCDHB16 |
| GO:0051928 | Positive regulation of calcium ion transport | 5.074 | 3.98E-03 | 9 | 136 | 6.62 | CX3CL1 CCL2 WFS1 SNCA AKAP6 STC1 LPAR3 RYR2 CCL5 |
| GO:0048870 | Cell motility | 1.814 | 4.05E-03 | 44 | 1776 | 2.48 | CX3CL1 DAPK2 DNAH5 GPC4 CXCL2 SLC7A8 CD40 BEX4 PLAT DOCK8 CCL2 TSPAN11 SOD2 CCL20 ITGB6 RND3 NR4A3 BMP2 CCR7 MGAT3 FGF13 GPNMB LEF1 ACKR3 CXCL14 NR4A2 F11R STC1 VCAM1 IGSF8 CXCL5 CXCL1 CXCL8 CMKLR1 ADGRB1 CAMK1D PROS1 CCK APOD SRGAP3 NTNG2 L1CAM MMP12 CCL5 |
| GO:0051674 | Localization of cell | 1.814 | 4.05E-03 | 44 | 1776 | 2.48 | CX3CL1 DAPK2 DNAH5 GPC4 CXCL2 SLC7A8 CD40 BEX4 PLAT DOCK8 CCL2 TSPAN11 SOD2 CCL20 ITGB6 RND3 NR4A3 BMP2 CCR7 MGAT3 FGF13 GPNMB LEF1 ACKR3 CXCL14 NR4A2 F11R STC1 VCAM1 IGSF8 CXCL5 CXCL1 CXCL8 CMKLR1 ADGRB1 CAMK1D PROS1 CCK APOD SRGAP3 NTNG2 L1CAM MMP12 CCL5 |
| GO:0003013 | Circulatory system process | 2.503 | 4.08E-03 | 22 | 632 | 3.48 | TMEM38A SLC7A8 PCSK5 RENBP SLC1A2 SOD2 STAT1 KCNJ2 ABCC4 FGF13 CD36 KCNK1 SLCO2B1 F11R STC1 TAC3 BDKRB2 KCNH6 SCN4B SCN5A RYR2 RASL10B |
| GO:0035725 | Sodium ion transmembrane transport | 4.480 | 4.26E-03 | 10 | 177 | 5.65 | SLC4A7 SLC6A15 SLC6A12 KLHL24 FGF13 KCNK1 STOM SCN4B SCN5A TPCN1 |
| GO:0034097 | Response to cytokine | 1.978 | 4.34E-03 | 35 | 1404 | 2.49 | CX3CL1 BIRC3 CXCL2 GGT1 SYNGR1 CD40 TNFSF13B RELB DOCK8 CCL2 SOD2 CCL20 STAT1 KYNU CCR7 LIF CHI3L1 CD36 LEF1 ACKR3 SNCA TSLP MMP3 AKAP6 HK2 SQSTM1 VCAM1 CXCL5 CXCL1 CXCL8 RAB43 CMKLR1 PRKN MMP12 CCL5 |
| GO:0042330 | Taxis | 2.481 | 4.39E-03 | 22 | 702 | 3.13 | CX3CL1 DAPK2 NOTCH3 RAP1GAP CXCL2 CCL2 CCL20 CCR7 GPNMB LEF1 ACKR3 CNTN4 CXCL14 VCAM1 CXCL5 CXCL1 CXCL8 CMKLR1 CAMK1D ROBO2 L1CAM CCL5 |
| GO:0050808 | Synapse organization | 2.788 | 4.55E-03 | 18 | 451 | 3.99 | CX3CL1 SDK2 GPC4 TANC1 PCDHB14 C3 FGF13 SNCA SDK1 SHANK1 SEZ6L2 PPFIA3 ADGRB1 ROBO2 NTNG2 L1CAM PCDHB16 ADGRL1 |
| GO:0034764 | Positive regulation of transmembrane transport | 3.740 | 4.60E-03 | 12 | 245 | 4.90 | CX3CL1 CCL2 SLC1A2 NR4A3 KCNJ2 C3 AZIN2 SNCA AKAP6 KCNJ6 CTSS RYR2 |
| GO:0099601 | Regulation of neurotransmitter receptor activity | 6.504 | 4.60E-03 | 7 | 88 | 7.95 | CCL2 KLHL24 DLG2 SHANK1 NPTX1 LYNX1 NPTXR |
| GO:0032101 | Regulation of response to external stimulus | 2.071 | 4.69E-03 | 31 | 1282 | 2.42 | ENPP4 CX3CL1 BIRC3 DAPK2 GGT1 PLAT RELB CCL2 DHX58 STAT1 GRK4 C3 CCR7 FGL2 CD36 HERC5 NFKBIZ SNCA TSLP CXCL14 MMP3 ADCY8 CXCL8 C1QTNF1 CMKLR1 CAMK1D PROS1 ROBO2 CCK MMP12 CCL5 |
| GO:0098662 | Inorganic cation transmembrane transport | 2.338 | 4.83E-03 | 24 | 843 | 2.85 | CX3CL1 SLC4A7 SLC6A15 TMEM38A SLC6A12 KLHL24 KCNJ2 CCR7 FGF13 KCNC3 KCNK1 SNCA ATP6V0D2 STOM AKAP6 KCNJ6 RRAD KCNH6 SCN4B SCN5A KCNK12 TPCN1 RYR2 |
| GO:0002684 | Positive regulation of immune system process | 2.168 | 6.04E-03 | 27 | 1190 | 2.27 | CFH CX3CL1 DAPK2 CD40 TNFSF13B RELB DOCK8 CCL2 DHX58 CCL20 IGFBP2 NR4A3 C3 CCR7 LIF CD36 LEF1 NFKBIZ TSLP CXCL14 VCAM1 CXCL8 CMKLR1 CAMK1D PROS1 MMP12 CCL5 |
| GO:0050865 | Regulation of cell activation | 2.570 | 7.42E-03 | 19 | 678 | 2.80 | CYP26B1 CX3CL1 CD40 TNFSF13B DOCK8 CCL2 IGFBP2 NR4A3 CCR7 FGL2 GPNMB LEF1 NFKBIZ SNCA TSLP F11R VCAM1 C1QTNF1 CCL5 |
| GO:0001504 | Neurotransmitter uptake | 9.292 | 7.61E-03 | 5 | 50 | 10.00 | SLC1A2 SLC6A12 SYNGR3 SNCA PRKN |
| GO:0031667 | Response to nutrient levels | 2.509 | 9.52E-03 | 19 | 530 | 3.58 | CYP26B1 PDK4 RRAGD CD40 LIPG SOD2 STAT1 IGFBP2 KYNU SGIP1 GDF15 GABARAPL1 CYP1A1 ATP6V0D2 SESN3 STC1 VCAM1 ATF3 CCK |
| GO:0042592 | Homeostatic process | 1.719 | 9.72E-03 | 44 | 1911 | 2.30 | CYP26B1 PDK4 CX3CL1 SLC4A7 CP LPAR2 TMEM38A AKR1B1 SLC7A8 CD40 CST4 LIPG TNFSF13B CCL2 WFS1 SOD2 ITGB6 STAT1 SGIP1 NR4A3 KCNJ2 CCR7 CD36 CTSK ACKR3 SNCA ATP6V0D2 SESN3 AKAP6 TMEM178A ADCY8 F11R STC1 HK2 BDKRB2 LPAR3 C1QTNF1 CMKLR1 SCN5A PRKN TPCN1 CCK RYR2 CCL5 |
| GO:0055082 | Cellular chemical homeostasis | 2.167 | 9.72E-03 | 25 | 882 | 2.83 | CX3CL1 SLC4A7 CP LPAR2 TMEM38A CD40 WFS1 KCNJ2 CCR7 CD36 ACKR3 SNCA ATP6V0D2 AKAP6 TMEM178A ADCY8 STC1 HK2 BDKRB2 LPAR3 C1QTNF1 CMKLR1 TPCN1 RYR2 CCL5 |
| GO:0043408 | Regulation of MAPK cascade | 2.363 | 9.72E-03 | 21 | 646 | 3.25 | CX3CL1 TENM1 CD40 CCL2 CCL20 DUSP4 BMP2 CCR7 LIF GDF15 CHI3L1 CD36 GPNMB ACKR3 ATF3 NEK10 SPRY1 LPAR3 C1QTNF1 PRKN CCL5 |
| GO:0032409 | Regulation of transporter activity | 3.182 | 9.72E-03 | 13 | 306 | 4.25 | CCL2 KLHL24 SYNGR3 FGF13 SNCA STOM DLG2 AKAP6 SHANK1 CTSS RRAD SCN4B RYR2 |
| GO:0086004 | Regulation of cardiac muscle cell contraction | 8.651 | 9.72E-03 | 5 | 34 | 14.71 | KCNJ2 FGF13 STC1 SCN5A RYR2 |
| GO:0035584 | Calcium-mediated signaling using intracellular calcium source | 12.544 | 9.72E-03 | 4 | 27 | 14.81 | CCL20 VCAM1 RYR2 ADGRL1 |
