# Supplementary material for: Mapping the core senescence phenotype of primary human colon fibroblasts
Source: Aging (Albany NY). 2024 Feb 21;16(4):3068–87. doi: 10.18632/aging.205577 (PMC10929841; doi:10.18632/aging.205577)
Supplement: Supplementary Tables 6A and 6B [file aging-16-205577-s006.docx]

**Supplemental Table 6A. GO analysis for biological processes on the Core senescence SASP subset (FDR ≤ 0.01).**

| **GO ID** | **Pathway** | **Fold enrichment** | **Enrichment FDR** | **N genes** | **Pathway genes** | **Gene ratio** | **Genes** |
| --- | --- | --- | --- | --- | --- | --- | --- |
| GO:0006954 | Inflammatory response | 7.790 | 1.66E-08 | 17 | 950 | 1.789 | CX3CL1 CXCL2 CD40 CCL2 CCL20 C3 BMP2 CHI3L1 SNCA TSLP MMP3 SCUBE1 CXCL5 CXCL1 CXCL8 APOD CCL5 |
| GO:0006959 | Humoral immune response | 22.910 | 1.66E-08 | 10 | 331 | 3.021 | CFH CXCL2 CCL2 C3 TSLP CXCL14 CXCL5 CXCL1 CXCL8 PROS1 |
| GO:0040011 | Locomotion (motility) | 4.053 | 2.79E-07 | 23 | 1982 | 1.160 | CX3CL1 GPC4 CXCL2 CD40 PLAT CCL2 COL1A1 CCL20 BMP2 FGF13 CNTN4 SNCA CXCL14 STC1 CXCL5 CXCL1 CXCL8 ADGRB1 PROS1 CCK APOD MMP12 CCL5 |
| GO:0016477 | Cell migration | 4.512 | 2.79E-07 | 21 | 1590 | 1.321 | CX3CL1 GPC4 CXCL2 CD40 PLAT CCL2 COL1A1 CCL20 BMP2 FGF13 CXCL14 STC1 CXCL5 CXCL1 CXCL8 ADGRB1 PROS1 CCK APOD MMP12 CCL5 |
| GO:0007267 | Cell-cell signaling | 4.118 | 9.93E-07 | 21 | 1885 | 1.114 | CX3CL1 GPC4 PCSK5 PLAT CCL2 COL1A1 CPE CCL20 IGFBP2 C3 BMP2 LIF FGF13 GDF15 CNTN4 SNCA CXCL14 CXCL5 C1QTNF1 ADGRB1 CCL5 |
| GO:0019730 | Antimicrobial humoral response | 34.038 | 2.50E-06 | 6 | 166 | 3.614 | CXCL2 TSLP CXCL14 CXCL5 CXCL1 CXCL8 |
| GO:0002684 | Positive regulation of immune system process | 5.718 | 2.81E-06 | 15 | 1190 | 1.261 | CFH CX3CL1 CD40 TNFSF13B CCL2 CCL20 IGFBP2 C3 LIF TSLP CXCL14 CXCL8 PROS1 MMP12 CCL5 |
| GO:0009607 | Response to biotic stimulus | 4.417 | 4.29E-06 | 18 | 1905 | 0.945 | CFH CX3CL1 CXCL2 CD40 CCL2 CCL20 C3 BMP2 FGL2 SNCA TSLP CXCL14 CXCL5 CXCL1 CXCL8 ADGRB1 MMP12 CCL5 |
| GO:0034097 | Response to cytokine | 4.567 | 6.26E-06 | 17 | 1404 | 1.211 | CX3CL1 CXCL2 CD40 TNFSF13B CCL2 COL1A1 CCL20 LIF CHI3L1 SNCA TSLP MMP3 CXCL5 CXCL1 CXCL8 MMP12 CCL5 |
| GO:0007186 | G protein-coupled receptor signaling pathway | 6.232 | 8.08E-06 | 13 | 1569 | 0.829 | CX3CL1 CXCL2 CCL2 CPE CCL20 C3 SNCA CXCL5 CXCL1 CXCL8 ADGRB1 CCK CCL5 |
| GO:0022617 | Extracellular matrix disassembly | 25.993 | 8.11E-06 | 6 | 94 | 6.383 | TLL2 CTSK MMP3 SCUBE1 CTSS MMP12 |
| GO:0060326 | Cell chemotaxis | 10.941 | 8.47E-06 | 9 | 343 | 2.624 | CX3CL1 CXCL2 CCL2 CCL20 CXCL14 CXCL5 CXCL1 CXCL8 CCL5 |
| GO:0022610 | Biological adhesion | 4.058 | 1.04E-05 | 18 | 1646 | 1.094 | CX3CL1 GPC4 TNFSF13B CCL2 COL1A1 IGFBP2 BMP2 FGL2 LIF CLCA2 CNTN4 SERPINI1 CXCL8 C1QTNF1 ADGRB1 APOD MMP12 CCL5 |
| GO:0071356 | Cellular response to tumor necrosis factor | 9.837 | 1.75E-05 | 9 | 373 | 2.413 | CX3CL1 CD40 TNFSF13B CCL2 COL1A1 CCL20 CHI3L1 CXCL8 CCL5 |
| GO:0032101 | Regulation of response to external stimulus | 4.759 | 1.79E-05 | 15 | 1282 | 1.170 | CX3CL1 PLAT CCL2 C3 FGL2 SNCA TSLP CXCL14 MMP3 CXCL8 C1QTNF1 PROS1 CCK MMP12 CCL5 |
| GO:0001775 | Cell activation | 4.091 | 1.96E-05 | 17 | 1658 | 1.025 | CX3CL1 CD40 TNFSF13B PLAT CCL2 COL1A1 IGFBP2 C3 FGL2 CHI3L1 SNCA TSLP CTSS CXCL1 CXCL8 C1QTNF1 CCL5 |
| GO:0070555 | Response to interleukin-1 | 11.623 | 2.10E-05 | 8 | 252 | 3.175 | CX3CL1 CD40 CCL2 CCL20 CHI3L1 SNCA CXCL8 CCL5 |
| GO:0030198 | Extracellular matrix organization | 7.309 | 4.25E-05 | 10 | 450 | 2.222 | TLL2 COL1A1 BMP2 CTSK MMP3 SCUBE1 CTSS SPINT1 HPSE2 MMP12 |
| GO:0042127 | Regulation of cell population proliferation | 3.589 | 4.39E-05 | 18 | 1817 | 0.991 | CX3CL1 CD40 TNFSF13B CCL2 IGFBP2 BMP2 LIF TSLP CXCL5 CXCL1 SPINT1 CXCL8 HPSE2 ADGRB1 CCK APOD MMP12 CCL5 |
| GO:0072359 | Circulatory system development | 4.132 | 7.97E-05 | 15 | 1127 | 1.331 | CX3CL1 PCSK5 CD40 CCL2 COL1A1 CPE C3 BMP2 LIF CHI3L1 SCUBE1 SPINT1 CXCL8 ADGRB1 APOD |
| GO:0071347 | Cellular response to interleukin-1 | 11.503 | 1.01E-04 | 7 | 225 | 3.111 | CX3CL1 CD40 CCL2 CCL20 CHI3L1 CXCL8 CCL5 |
| GO:0001934 | Positive regulation of protein phosphorylation | 5.152 | 1.07E-04 | 12 | 767 | 1.565 | CX3CL1 CD40 C3 BMP2 LIF FGF13 GDF15 CHI3L1 SNCA TSLP CCK CCL5 |
| GO:0001944 | Vasculature development | 4.913 | 1.69E-04 | 12 | 748 | 1.604 | CX3CL1 PCSK5 CD40 CCL2 COL1A1 C3 LIF CHI3L1 SPINT1 CXCL8 ADGRB1 APOD |
| GO:0046903 | Secretion | 3.580 | 1.94E-04 | 16 | 1636 | 0.978 | PCSK5 C3 BMP2 FGL2 LIF CHI3L1 PCSK6 SNCA STC1 CTSS CXCL1 C1QTNF1 PROS1 CCK SELENOP CCL5 |
| GO:0051336 | Regulation of hydrolase activity | 3.654 | 3.14E-04 | 15 | 1418 | 1.058 | CX3CL1 PCSK5 CD40 CST4 CCL2 CCL20 C3 BMP2 PCSK6 SNCA SERPINI1 SPINT1 PROS1 CCK CCL5 |
| GO:0051241 | Negative regulation of multicellular organismal process | 4.220 | 3.14E-04 | 13 | 1111 | 1.170 | CX3CL1 PLAT BMP2 FGL2 FGF13 GDF15 CTSK STC1 C1QTNF1 ADGRB1 PROS1 CCK APOD |
| GO:1901700 | Response to oxygen-containing compound | 3.215 | 3.43E-04 | 17 | 1832 | 0.928 | CX3CL1 CXCL2 CD40 CCL2 COL1A1 IGFBP2 GDF15 SNCA MMP3 STC1 CXCL5 CXCL1 CXCL8 APOD AKR1B10 MMP12 CCL5 |
| GO:0010817 | Regulation of hormone levels | 6.198 | 4.14E-04 | 9 | 581 | 1.549 | TG PCSK5 BMP2 LIF PCSK6 CTSK C1QTNF1 AKR1B10 CCL5 |
| GO:0140352 | Export from cell | 3.507 | 4.27E-04 | 15 | 1545 | 0.971 | PCSK5 C3 BMP2 FGL2 LIF CHI3L1 PCSK6 SNCA CTSS CXCL1 C1QTNF1 PROS1 CCK SELENOP CCL5 |
| GO:0033993 | Response to lipid | 4.352 | 4.27E-04 | 12 | 1017 | 1.180 | CX3CL1 CXCL2 CD40 CCL2 COL1A1 IGFBP2 SNCA STC1 CXCL5 CXCL1 CXCL8 CCL5 |
| GO:0042330 | Taxis | 5.354 | 4.27E-04 | 10 | 702 | 1.425 | CX3CL1 CXCL2 CCL2 CCL20 CNTN4 CXCL14 CXCL5 CXCL1 CXCL8 CCL5 |
| GO:1901701 | Cellular response to oxygen-containing compound | 3.711 | 4.38E-04 | 14 | 1313 | 1.066 | CX3CL1 CXCL2 CD40 CCL2 COL1A1 GDF15 SNCA MMP3 STC1 CXCL5 CXCL1 CXCL8 AKR1B10 CCL5 |
| GO:0043491 | Protein kinase B signaling | 8.642 | 4.52E-04 | 7 | 278 | 2.518 | CX3CL1 CD40 CCL2 GDF15 CHI3L1 C1QTNF1 CCL5 |
| GO:0010562 | Positive regulation of phosphorus metabolic process | 4.255 | 4.86E-04 | 12 | 942 | 1.274 | CX3CL1 CD40 C3 BMP2 LIF FGF13 GDF15 CHI3L1 SNCA TSLP CCK CCL5 |
| GO:0051270 | Regulation of cellular component movement | 3.877 | 5.31E-04 | 13 | 1092 | 1.190 | CX3CL1 CD40 CCL2 COL1A1 CCL20 BMP2 FGF13 CXCL14 STC1 CXCL8 ADGRB1 APOD CCL5 |
| GO:0034341 | Response to interferon-gamma | 10.669 | 5.31E-04 | 6 | 312 | 1.923 | CX3CL1 CD40 CCL2 CCL20 SNCA CCL5 |
| GO:0032963 | Collagen metabolic process | 14.708 | 5.74E-04 | 5 | 113 | 4.425 | COL1A1 CTSK MMP3 CTSS MMP12 |
| GO:0098542 | Defense response to other organism | 4.156 | 5.76E-04 | 12 | 1499 | 0.801 | CFH CX3CL1 CD40 CCL2 CCL20 C3 FGL2 SNCA TSLP ADGRB1 MMP12 CCL5 |
| GO:0035295 | Tube development | 3.773 | 6.51E-04 | 13 | 1094 | 1.188 | CX3CL1 PCSK5 CD40 CCL2 C3 BMP2 LIF CHI3L1 SPINT1 CXCL8 ADGRB1 APOD MMP12 |
| GO:0042445 | Hormone metabolic process | 10.139 | 6.51E-04 | 6 | 259 | 2.317 | TG PCSK5 BMP2 PCSK6 CTSK AKR1B10 |
| GO:0048646 | Anatomical structure formation involved in morphogenesis | 3.745 | 6.82E-04 | 13 | 1183 | 1.099 | CX3CL1 CD40 TNFSF13B CCL2 COL1A1 C3 BMP2 GDF15 CHI3L1 SPINT1 CXCL8 ADGRB1 APOD |
| GO:0070371 | ERK1 and ERK2 cascade | 7.758 | 7.16E-04 | 7 | 350 | 2.000 | CX3CL1 CCL2 CCL20 BMP2 LIF CHI3L1 CCL5 |
| GO:0051928 | Positive regulation of calcium ion transport | 13.386 | 7.96E-04 | 5 | 136 | 3.676 | CX3CL1 CCL2 SNCA STC1 CCL5 |
| GO:0032102 | Negative regulation of response to external stimulus | 6.109 | 9.91E-04 | 8 | 581 | 1.377 | CX3CL1 PLAT CCL2 FGL2 C1QTNF1 PROS1 CCK MMP12 |
| GO:2000145 | Regulation of cell motility | 3.864 | 0.001017 | 12 | 1010 | 1.188 | CX3CL1 CD40 CCL2 COL1A1 CCL20 BMP2 CXCL14 STC1 CXCL8 ADGRB1 APOD CCL5 |
| GO:0045861 | Negative regulation of proteolysis | 7.252 | 0.00103 | 7 | 385 | 1.818 | CST4 PLAT C3 SNCA SERPINI1 SPINT1 PROS1 |
| GO:0009967 | Positive regulation of signal transduction | 3.116 | 0.001111 | 15 | 1644 | 0.912 | CX3CL1 CD40 CCL2 COL1A1 CCL20 C3 BMP2 LIF GDF15 CHI3L1 TSLP SCUBE1 C1QTNF1 MMP12 CCL5 |
| GO:0031401 | Positive regulation of protein modification process | 3.752 | 0.001248 | 12 | 1018 | 1.179 | CX3CL1 CD40 C3 BMP2 LIF FGF13 GDF15 CHI3L1 SNCA TSLP CCK CCL5 |
| GO:0051240 | Positive regulation of multicellular organismal process | 3.229 | 0.001383 | 14 | 1614 | 0.867 | CX3CL1 CD40 COL1A1 C3 BMP2 LIF CHI3L1 TSLP SPINT1 CXCL8 C1QTNF1 ADGRB1 CCK MMP12 |
| GO:0022603 | Regulation of anatomical structure morphogenesis | 3.694 | 0.001393 | 12 | 1034 | 1.161 | CX3CL1 GPC4 CD40 TNFSF13B CCL2 C3 BMP2 LIF FGF13 CHI3L1 CXCL8 ADGRB1 |
| GO:0000165 | MAPK cascade | 3.941 | 0.001624 | 11 | 944 | 1.165 | CX3CL1 CD40 CCL2 CCL20 BMP2 LIF FGF13 GDF15 CHI3L1 C1QTNF1 CCL5 |
| GO:0032103 | Positive regulation of response to external stimulus | 5.557 | 0.001654 | 8 | 622 | 1.286 | CX3CL1 C3 SNCA TSLP CXCL14 CXCL8 MMP12 CCL5 |
| GO:0032455 | Nerve growth factor processing | 119.134 | 0.001975 | 2 | 4 | 50.000 | PCSK5 PCSK6 |
| GO:0045087 | Innate immune response | 4.210 | 0.00202 | 10 | 1258 | 0.795 | CFH CX3CL1 CD40 CCL2 CCL20 C3 SNCA ADGRB1 MMP12 CCL5 |
| GO:0097696 | Receptor signaling pathway via STAT | 10.543 | 0.002033 | 5 | 177 | 2.825 | CD40 CCL2 LIF TSLP CCL5 |
| GO:0071774 | Response to fibroblast growth factor | 10.182 | 0.00236 | 5 | 147 | 3.401 | CCL2 COL1A1 SNCA CXCL8 CCL5 |
| GO:0009611 | Response to wounding | 4.505 | 0.002699 | 9 | 718 | 1.253 | CX3CL1 CD40 PLAT COL1A1 SCUBE1 C1QTNF1 PROS1 APOD MMP12 |
| GO:0060191 | Regulation of lipase activity | 14.663 | 0.002826 | 4 | 101 | 3.960 | PCSK5 PCSK6 SNCA CCL5 |
| GO:0022411 | Cellular component disassembly | 4.440 | 0.002969 | 9 | 615 | 1.463 | CX3CL1 TLL2 C3 FGF13 CTSK MMP3 SCUBE1 CTSS MMP12 |
| GO:0098883 | Synapse pruning | 95.307 | 0.003058 | 2 | 11 | 18.182 | CX3CL1 C3 |
| GO:2000026 | Regulation of multicellular organismal development | 3.122 | 0.003177 | 13 | 1438 | 0.904 | CX3CL1 TG CD40 C3 BMP2 FGL2 LIF FGF13 CHI3L1 CTSK SPINT1 CXCL8 ADGRB1 |
| GO:0097278 | Complement-dependent cytotoxicity | 79.423 | 0.004164 | 2 | 10 | 20.000 | CFH C3 |
| GO:2000427 | Positive regulation of apoptotic cell clearance | 79.423 | 0.004164 | 2 | 20 | 10.000 | CCL2 C3 |
| GO:0052547 | Regulation of peptidase activity | 5.245 | 0.005348 | 7 | 512 | 1.367 | CST4 C3 SNCA SERPINI1 SPINT1 PROS1 CCK |
| GO:0001957 | Intramembranous ossification | 68.077 | 0.005419 | 2 | 7 | 28.571 | COL1A1 CTSK |
| GO:0045687 | Positive regulation of glial cell differentiation | 21.024 | 0.005741 | 3 | 42 | 7.143 | BMP2 LIF SPINT1 |
| GO:0051216 | Cartilage development | 7.942 | 0.005966 | 5 | 202 | 2.475 | COL1A1 BMP2 CHI3L1 CTSK STC1 |
| GO:0035239 | Tube morphogenesis | 3.567 | 0.005968 | 10 | 886 | 1.129 | CX3CL1 CD40 CCL2 C3 BMP2 CHI3L1 SPINT1 CXCL8 ADGRB1 APOD |
| GO:0006956 | Complement activation | 20.423 | 0.006064 | 3 | 95 | 3.158 | CFH C3 PROS1 |
| GO:2000147 | Positive regulation of cell motility | 4.392 | 0.006098 | 8 | 581 | 1.377 | CX3CL1 CD40 COL1A1 CCL20 BMP2 CXCL14 CXCL8 CCL5 |
| GO:0050729 | Positive regulation of inflammatory response | 11.213 | 0.006197 | 4 | 158 | 2.532 | CX3CL1 C3 SNCA TSLP |
| GO:0050817 | Coagulation | 6.058 | 0.00627 | 6 | 378 | 1.587 | CD40 PLAT COL1A1 SCUBE1 C1QTNF1 PROS1 |
| GO:0016322 | Neuron remodeling | 59.567 | 0.006558 | 2 | 14 | 14.286 | CX3CL1 C3 |
| GO:2000425 | Regulation of apoptotic cell clearance | 59.567 | 0.006558 | 2 | 22 | 9.091 | CCL2 C3 |
| GO:0071363 | Cellular response to growth factor stimulus | 3.795 | 0.00706 | 9 | 722 | 1.247 | CCL2 COL1A1 BMP2 GDF15 PCSK6 SNCA SPINT1 CXCL8 CCL5 |
| GO:0043277 | Apoptotic cell clearance | 18.811 | 0.007124 | 3 | 64 | 4.688 | CCL2 C3 ADGRB1 |
| GO:0043269 | Regulation of ion transport | 4.189 | 0.007633 | 8 | 763 | 1.048 | CX3CL1 CCL2 FGF13 SNCA STC1 CTSS CCK CCL5 |
| GO:0010628 | Positive regulation of gene expression | 3.143 | 0.007724 | 11 | 1336 | 0.823 | CX3CL1 CD40 C3 BMP2 LIF CHI3L1 TSLP CXCL8 C1QTNF1 MMP12 CCL5 |
| GO:0051960 | Regulation of nervous system development | 4.793 | 0.007739 | 7 | 461 | 1.518 | CX3CL1 TG BMP2 LIF FGF13 SPINT1 ADGRB1 |
| GO:0014048 | Regulation of glutamate secretion | 52.948 | 0.007837 | 2 | 16 | 12.500 | SNCA CCK |
| GO:1901741 | Positive regulation of myoblast fusion | 52.948 | 0.007837 | 2 | 20 | 10.000 | GDF15 ADGRB1 |
| GO:0007566 | Embryo implantation | 17.870 | 0.007905 | 3 | 52 | 5.769 | PCSK5 LIF STC1 |
| GO:0070848 | Response to growth factor | 3.672 | 0.008373 | 9 | 750 | 1.200 | CCL2 COL1A1 BMP2 GDF15 PCSK6 SNCA SPINT1 CXCL8 CCL5 |
| GO:0051050 | Positive regulation of transport | 3.337 | 0.008571 | 10 | 1013 | 0.987 | CX3CL1 CCL2 C3 BMP2 SNCA STC1 CTSS C1QTNF1 CCK CCL5 |
| GO:1900047 | Negative regulation of hemostasis | 17.019 | 0.008809 | 3 | 57 | 5.263 | PLAT C1QTNF1 PROS1 |
| GO:1902533 | Positive regulation of intracellular signal transduction | 3.309 | 0.008963 | 10 | 1028 | 0.973 | CX3CL1 CD40 CCL2 CCL20 BMP2 LIF GDF15 CHI3L1 C1QTNF1 CCL5 |
| GO:0016485 | Protein processing | 6.926 | 0.009188 | 5 | 252 | 1.984 | PCSK5 PLAT CPE PCSK6 CTSS |

**Supplemental Table 6B. GO analysis for molecular function on the Core senescence SASP subset (FDR ≤ 0.05).**

| **Pathway** | **Fold Enrichment** | **Enrichment FDR** | **N Genes** | **Pathway Genes** | **Gene ratio** | **Genes** |
| --- | --- | --- | --- | --- | --- | --- |
| Chemokine/Cytokine receptor binding | 6.013 | 3.33E-12 | 24 | 1722 | 1.394 | CX3CL1 TG CXCL2 TNFSF13B PLAT CCL2 CPE CCL20 IGFBP2 C3 BMP2 FGL2 LIF FGF13 GDF15 TSLP CXCL14 STC1 CXCL5 CXCL1 CXCL8 CDNF CCK CCL5 |
| Peptidase activity | 7.464 | 2.34E-07 | 13 | 711 | 1.828 | ADAM28 TLL2 PCSK5 PLAT CPE FGL2 CLCA2 PCSK6 CTSK PAMR1 MMP3 CTSS MMP12 |
| Growth factor activity | 14.738 | 4.40E-05 | 6 | 165 | 3.636 | BMP2 LIF FGF13 GDF15 CXCL1 CDNF |
| Proteoglycan binding | 32.865 | 7.37E-05 | 4 | 38 | 10.526 | CFH CTSK CTSS HPSE2 |
| Interleukin-8 receptor binding | 238.268 | 1.98E-04 | 2 | 3 | 66.667 | CX3CL1 CXCL8 |
| Metallopeptidase activity | 10.285 | 2.51E-04 | 6 | 205 | 2.927 | ADAM28 TLL2 CPE CLCA2 MMP3 MMP12 |
| Collagen binding | 15.624 | 0.001198997 | 4 | 76 | 5.263 | CTSK CTSS C1QTNF1 MMP12 |
| Serine-type endopeptidase activity | 13.056 | 0.002304401 | 4 | 195 | 2.051 | PCSK5 PLAT PCSK6 PAMR1 |
| BMP receptor binding | 47.654 | 0.006123589 | 2 | 13 | 15.385 | BMP2 GDF15 |
| Hormone activity | 15.209 | 0.007720572 | 3 | 130 | 2.308 | BMP2 GDF15 |
| Heparan sulfate proteoglycan binding | 39.711 | 0.008316907 | 2 | 18 | 11.111 | CFH HPSE2 |
